# Dose–structure–population interactions of protein supplementation combined with resistance training on body composition: a Bayesian network meta-analysis with dose–response modeling

**DOI:** 10.3389/fnut.2026.1860234

**Published:** 2026-08-06

**Authors:** Yunxia Li, Gang Qin, Ziyu Wang, Jinghao Zhang

**Affiliations:** 1 School of Physical Education, Shandong Sport University, Rizhao, Shandong, China; 2 College of Sports, Hanyang University, Seoul, Republic of Korea; 3 College of Sports, Sejong University, Seoul, Republic of Korea; 4 Physical Education Department, Shandong Technology and Business University, Yantai, Shandong, China

**Keywords:** Bayesian, dose–response, lean body mass, network meta-analysis, protein supplementation, resistance training, SUCRA, whey protein

## Abstract

**Background:**

Protein supplementation is frequently utilized in conjunction with resistance training for the purpose of enhancing muscle mass and strength. Nevertheless, the efficacy of different protein types, dosing thresholds, and population-specific effects is debatable.

**Methods:**

A systematic search was performed on PubMed, Web of Science, Scopus, SPORTDiscus, Cochrane CENTRAL, EMBASE databases through February 2026. RCTs comparing protein supplements (whey, casein, soy, pea, rice, milk, or blend) with placebo during supervised RT (≥6 weeks) were eligible. A Bayesian random-effects NMA was conducted using gemtc and JAGS. Dose–response curves were fitted with restricted cubic splines. The protocol was registered on PROSPERO (CRD420261340515).

**Results:**

Thirty-six RCTs (1,552 participants; 89 trial arms) formed a fully connected network of eight nodes. For LBM, whey protein ranked first (SUCRA = 0.91; MD vs. placebo: +1.24 kg, 95% CrI 0.61–1.87), followed by milk (SUCRA = 0.78; +1.18 kg) and casein (SUCRA = 0.72; +0.92 kg). The dose–response curve for whey indicated a plateau at 30–35 g/d. In older adults (≥65 years), the superiority of whey narrowed (SUCRA = 0.89 vs. 0.92 in younger adults), while casein’s SUCRA increased from 0.68 in younger adults to 0.76 in older adults. However, this subgroup analysis was based on only nine trials, and the observed ranking changes may be unstable. Model convergence was satisfactory ( R^^ = 1.01; ESS = 3,420), and node-splitting detected no significant inconsistency (all *p* > 0.10).

**Conclusion:**

Among commonly available protein supplements, whey protein at approximately 30–35 g/d was associated with the largest gains in lean body mass when combined with RT, although this dose–response estimate is exploratory. Older adults may benefit from casein- or mixed-protein strategies as the advantage of whey is diminished in this population. Plant-based proteins showed smaller effect estimates than dairy-derived proteins, but the evidence certainty was low to very low and the credible intervals were wide, limiting firm conclusions.

**Systematic review registration:**

Identifier: CRD420261340515.

## 1 Introduction

Resistance training and protein ingestion are considered to be the foundation of all interventions aiming to build skeletal muscle mass and strength throughout life (1, 2). Muscle protein synthesis (MPS), which is the biochemical basis of skeletal muscle hypertrophy, is an acute response to mechanical stimuli and amino acid availability, especially leucine, which is a potent activator of the mechanistic target of rapamycin complex 1 (mTORC1) signaling pathway (3, 4). The current recommendations from the International Society of Sports Nutrition (ISSN) and the American College of Sports Medicine (ACSM) for total daily protein intake are 1.4–2.0 g/kg/d for individuals engaging in regular RT. However, these recommendations view different sources of dietary protein as similar (5). In practical terms, athletes and recreational exercisers can choose from whey, casein, soy, pea, rice, and various blended types, all of which vary in terms of amino acid content, digestion rate, and cost.

Despite the large body of research, three gaps exist in the literature. First, the majority of RCTs and subsequent meta-analyses have utilized pairwise designs, where the intervention is compared against placebo or another protein intervention individually (6, 7). A previous meta-analysis, pooling data from 22 RCTs, confirmed protein supplementation increases lean body mass (LBM) during RT but did not differentiate between protein types (6). A subsequent review included 49 RCTs and yielded a similar conclusion, observing a dose–response relationship without specifying the optima for different types (7). More recent inclusions have seen a meta-analysis conducted on whey protein in the context of a resistance-trained population; however, the scope of this meta-analysis was narrow in its inclusion criteria for a single protein source (8). A comprehensive network meta-analysis, which simultaneously anchors different protein classifications on a common efficacy continuum, has been notably absent. Second, the dose–response relationship has been defined only in broad terms. Most reviews have divided the dose–response relationship into “high versus low” doses or have arbitrarily defined different thresholds (e.g., 20 g vs. 40 g per serving) without fitting continuous dose–response curves with the capacity to detect an inflection point (9). This is clinically relevant because, for instance, while whey protein appears to plateau at 30 g/d, muscle protein synthesis continues to be elevated by soy protein until 45 g/d, possibly because of differences in leucine density and digestibility. Moreover, the relationship between population factors and the efficacy of supplementation has yet to be explored. In older populations, anabolic resistance requires a higher amount of leucine per meal (~2.5–3 g) to produce equivalent MPS rates (10, 11). It is unclear whether these differential effects exist between types of proteins at the meta-analytic level. In addition, training status (experienced lifters vs. novices) and sex may impact the degree of adaptation, although these are rarely quantified beyond general discussion.

Bayesian NMA offers a way to address the first gap in a principled manner. In this approach, information from indirect comparisons is used to complement direct comparisons in a way that handles the imbalance in head-to-head comparisons (whey is far more studied than rice or pea) more smoothly than the frequentist approach, in which whey is much better represented than rice or pea (12, 13). With the inclusion of restricted cubic splines in the model for dose–response and subgroup analyses, all three gaps can be addressed in a single framework.

Accordingly, this study pursued four objectives: (a) to rank seven protein supplement types and placebo for their effects on LBM and muscular strength during RT of at least 6 weeks, using Bayesian NMA with SUCRA; (b) to model non-linear dose–response curves for each protein source and identify the dose at which LBM gains plateau; (c) to examine whether age (≥65 vs. <65 years) and training status modify the relative treatment effects; and (d) to test whether protein type, dose, and population characteristics interact in a clinically meaningful way.

## 2 Methods

### 2.1 Protocol and registration

This systematic review was prospectively registered with PROSPERO (CRD420261340515) and conducted in accordance with the Preferred Reporting Items for Systematic Reviews and Meta-Analyses for Network Meta-Analyses (PRISMA-NMA) statement (14). No deviations from the registered protocol occurred. A completed PRISMA-NMA checklist is available in Supplementary Table S1.

### 2.2 Eligibility criteria

Studies were included if they met the following PICOS criteria: Population. Healthy adults aged 18 years or older, including overweight or obese individuals. Trials involving patients with chronic diseases that are known to affect muscle protein metabolism (e.g., cancer cachexia, chronic kidney disease) were excluded.

Intervention. Oral protein supplementation in the form of whey (isolated, concentrate, hydrolyzed, or native), casein (micellar or calcium caseinate), soy (isolated or concentrate), pea, rice, milk (whole or skim), or a specific combination of two or more types, taken in association with a supervised plan of resistance training for a minimum of 6 weeks and a minimum two sessions per week. The range for daily protein supplementation varied between 15 and 75 g/d.

Comparator. Placebo (carbohydrate-matched, isocaloric, or inert), no supplementation, or a different protein type.

Outcomes. The main outcomes were: (a) change in lean body mass (LBM) or fat-free mass (FFM) using dual-energy X-ray absorptiometry (DXA), bioelectrical impedance analysis, or hydrostatic weighing; and (b) change in maximal dynamic strength, quantified as the one-repetition maximum (1-RM) for any compound exercise. Secondary outcomes were body fat percentage, muscle thickness, and muscle cross-sectional area (CSA).

Study design. Only parallel-group RCTs were eligible. Crossover trials were excluded due to potential carryover effects on body composition.

### 2.3 Information sources and search strategy

This review was undertaken by searching six electronic databases from their inception to February 2026. These databases were: PubMed/MEDLINE, Web of Science Core Collection, Scopus, SPORTDiscus (via EBSCOhost), Cochrane Central Register of Controlled Trials (CENTRAL), and EMBASE. The search strategy consisted of the combination of three concept blocks employing Boolean operators: (1) terms related to protein supplementation (MeSH and free-text terms: “dietary proteins,” “whey proteins,” “caseins,” “soybean proteins,” “protein supplement,” “whey isolate,” “pea protein,” “rice protein,” “BCAA,” and “post-exercise protein”); (2) terms related to resistance training (“resistance training,” “weight lifting,” “strength training,” “progressive resistance,” “squat,” and “bench press”); and (3) terms for outcome measures (“muscle strength,” “body composition,” “lean body mass,” “fat-free mass,” “1RM,” and “muscle hypertrophy”). A study design filter was employed to restrict the search to RCTs. The search strings employed to search each database are provided in Supplementary file S1. The reference lists of all included studies and relevant narrative reviews were manually screened to identify additional trials. No language restriction was applied to the database searches. However, only English-language full-text articles were eligible for inclusion, which may have introduced language bias (see Limitations).

### 2.4 Study selection and data extraction

Two reviewers independently screened titles and abstracts, and then screened articles that were potentially relevant for inclusion. Disagreements were resolved through consensus or by a third reviewer. Quantification of inter-rater reliability was conducted by calculating Cohen’s kappa.

Data were extracted into a pre-piloted spreadsheet comprising six worksheets: study characteristics (author, year, country, design, sample size, age, sex distribution, training status, body mass index), intervention details (protein type, dose in g/d, frequency, timing relative to exercise, RT program parameters, duration), primary outcome data (LBM and 1-RM: pre- and post-intervention means, standard deviations, and change scores) (LBM and fat-free mass [FFM] are used interchangeably in this review), secondary outcome data, dose–response data (grouped by protein type and dose level), and extraction instructions. When studies reported standard errors or 95% confidence intervals instead of standard deviations, conversions were performed using established formulae (SD = SE × √*n*; SD = √*n* × (upper − lower)/3.92). If only pre- and post-intervention SDs were available without a change-score SD, the latter was imputed assuming a correlation of *r* = 0.5 between measurements (15). Medians and interquartile ranges were converted to means and SDs using an established method (16). All protein doses were standardized to g/d. When studies reported doses as g/kg/d, the mean body weight reported in each study was used for conversion. Only supplemental protein doses were extracted; total daily dietary protein intake was not included unless the study specifically quantified the supplemental component.

### 2.5 Risk of Bias and certainty of evidence

Methodological quality was assessed with the Cochrane Risk of Bias 2.0 (RoB 2.0) tool across five domains: randomization process, deviations from intended interventions, missing outcome data, outcome measurement, and selective reporting (17). Each domain was rated as “low risk,” “some concerns,” or “high risk.” Traffic-light plots and summary bar charts were generated with robvis (18). The certainty of evidence for key comparisons was evaluated using the Confidence in Network Meta-Analysis (CINeMA) framework, which appraises six domains: within-study bias, reporting bias, indirectness, imprecision, heterogeneity, and incoherence (19). A clinically important threshold of 0.5 kg for LBM was used to assess imprecision; comparisons whose 95% CrI included zero were rated as major concern. Reporting bias was assessed using the comparison-adjusted funnel plot and Egger’s regression test for comparisons with ten or more contributing studies. The CINeMA assessment was performed for all comparisons against placebo and for selected head-to-head comparisons (whey vs. casein, whey vs. soy, whey vs. pea); results are reported in Table 1 and Supplementary Table S2.

**Table 1:** CINEMA evidence certainty.

Comparison	Bias	Report	Indirect	Imprec	Hetero	Incoher	Overall
Whey vs. PBO	◑	○	○	○	◑	○	Moderate
Milk vs. PBO	○	○	○	●	◑	○	Low
Casein vs. PBO	◑	○	◑	◑	◑	○	Low
Soy vs. PBO	○	○	○	●	◑	○	Low
Pea vs. PBO	○	◑	○	●	◑	○	Very Low
Rice vs. PBO	◑	◑	◑	●	●	○	Very Low
Blend vs. PBO	○	◑	◑	●	●	○	Very Low

○ = No concern; ◑ = Some concerns; ● = Major concern.

### 2.6 Bayesian network Meta-analysis

Treatments were coded into eight nodes: whey, casein, soy, pea, rice, milk, blend, and placebo/control. Multi-arm studies contributing more than one arm to the same node were collapsed to avoid double-counting. The analysis was performed in R 4.3.2, with the gemtc package (version 1.0–2) combined with JAGS 4.3.1 (20, 21).

The model was a Bayesian random effects consistency model with normal likelihood and identity link function, which is suited for continuous outcomes measured as mean difference. Vague prior distributions were provided in the form of a normal distribution *N* (0, 100^2^) for treatment effect parameters and a Uniform (0, 5) for the heterogeneity standard deviation (*τ*). Four Markov chain Monte Carlo (MCMC) algorithms were run in parallel, with 50,000 burn-in iterations and 100,000 sampling iterations for each algorithm, and a thinning interval of 10 iterations, yielding 40,000 posterior samples for each parameter.

Convergence was checked using the Gelman-Rubin diagnostic statistic ( R^^ < 1.05 for satisfactory convergence) together with visual inspection of trace plots. The effective sample size (ESS) had to be greater than 1,000 for all parameters.

Treatment rankings were quantified as the surface under the cumulative ranking curve (SUCRA), which varies between 0 (worst) and 1 (best) (22). Rankograms displaying the probability of each treatment occupying each rank complemented the SUCRA values. To triangulate the Bayesian rankings, frequentist P-scores were computed via the netmeta package and compared against SUCRA values (23).

### 2.7 Inconsistency and model fit

Local inconsistency was examined via the node-splitting method, which decomposes each closed loop into direct and indirect estimates and tests their agreement using a Bayesian *p*-value (24). Results are presented in Tables 2, 3. Global model fit was compared between the consistency model and an unrelated mean effects (UME) model using the deviance information criterion (DIC); a difference of ≥5 points in favor of consistency was taken as adequate support for the assumption (25). The random-effects model was also compared against a fixed-effect specification on DIC to confirm the appropriateness of modeling between-study variability. All DIC comparisons are summarized in Table 4. A contribution matrix was computed to quantify the percentage contribution of each direct comparison to each network estimate (Supplementary Figure S4) (26). A net heat plot was also generated to visualize the distribution of inconsistency across the network (Supplementary Figure S5) (27).

**Table 2:** SUCRA rankings by subgroup (LBM).

Treatment	Overall	Young	Older	Trained	Untrained
Whey	0.91	0.92	0.89	0.90	0.88
Milk	0.78	0.75	0.80	0.65	0.74
Casein	0.72	0.68	0.76	0.72	0.70
Rice	0.65	0.63	NE	0.60	NE
Blend	0.48	0.46	NE	NE	0.45
Soy	0.40	0.42	0.38	0.40	0.41
Pea	0.35	0.36	0.32	0.35	0.36
Placebo	0.01	0.02	0.01	0.01	0.02

NE, not estimable.

**Table 3:** Bayesian meta-regression coefficients

Covariate	*β*	95% CrI	Interpretation
Dose (per g/d)	0.024	0.004–0.044	+10 g/d → +0.24 kg LBM
Age (per year)	−0.008	−0.021–0.005	Trend (NS)
Duration (per wk)	0.035	0.002–0.068	Longer → greater LBM

**Table 4:** Node-splitting inconsistency assessment (LBM).

Comparison	Direct MD	Indirect MD	Diff	*p*
Whey vs. PBO	1.21 [0.55,1.87]	1.35 [0.42,2.28]	−0.14	0.78
Whey vs. Soy	0.72 [−0.08,1.52]	0.58 [−0.45,1.61]	0.14	0.82
Whey vs. Casein	0.35 [−0.55,1.25]	0.28 [−0.72,1.28]	0.07	0.91
Whey vs. Pea	0.80 [−0.05,1.65]	0.68 [−0.38,1.74]	0.12	0.85
Casein vs. PBO	0.88 [0.10,1.66]	1.02 [−0.12,2.16]	−0.14	0.83
Soy vs. PBO	0.50 [−0.25,1.25]	0.64 [−0.32,1.60]	−0.14	0.80
Milk vs. PBO	1.22 [0.15,2.29]	1.10 [−0.15,2.35]	0.12	0.88
Whey vs. Rice	0.30 [−0.88,1.48]	0.24 [−1.05,1.53]	0.06	0.94

All *p* > 0.10.

### 2.8 Dose–response modeling

Non-linear dose–response relationships were modeled using restricted cubic splines (RCS) with three knots placed at the 10th, 50th, and 90th percentiles of the observed dose distribution, implemented via the dosresmeta (version 2.0.1) and rms (version 6.7–0) R packages (28, 29). For protein types with four or more distinct dose levels, the full spline model was fitted; for types with fewer dose levels, a linear dose–response model served as a fallback. Predicted effects were generated across the range of 10–80 g/d. The plateau dose was defined as the inflection point at which the first derivative of the fitted curve approached zero --that is, the dose beyond which additional supplementation conferred negligible further benefit. Age-stratified dose–response curves were also fitted to explore whether older and younger adults exhibited different plateau thresholds (Supplementary Figure S7).

### 2.9 Subgroup analyses and Meta-regression

Two pre-specified subgroup analyses were conducted by fitting separate NMAs to (a) younger adults (<65 years) and (b) older adults (≥65 years), and to (a) trained individuals (≥2 years RT experience) and (b) untrained or sedentary participants. SUCRA values from each subgroup NMA were compared descriptively.

Bayesian meta-regression was used to extend the main network meta-analysis (NMA) by adding study-level covariates. These covariates were mean protein dose (g/d), mean participant age (years), and intervention duration (weeks). Regression coefficients (*β*) and 95% credible intervals (CrI) were presented. If the 95% CrI did not cover 0, the coefficient was considered to be statistically credible.

### 2.10 Cluster ranking analysis

To evaluate the performance of treatments on all these outcomes collectively, a cluster ranking plot was developed by plotting SUCRA results for LBM (x-axis) and those for muscular strength (y-axis) (22). This plot allows for the identification of treatments that perform well on both outcomes together and those that perform well on one outcome but poorly on another.

### 2.11 Publication Bias

A comparison-adjusted funnel plot was used to detect small study effects in the network (30). In addition, Egger’s regression test was used for pairwise comparisons supported by ten or more studies (31).

### 2.12 Sensitivity analyses

Five sensitivity analyses were performed to assess the robustness of the results obtained in the primary analysis: (1) removal of studies with high risk of bias, (2) restriction to double-blind RCTs, (3) replacement of the vague Uniform(0, 5) prior for *τ* with an informative prior, a half-normal distribution with SD = 1, (4) leave-one-out analysis to evaluate the effect of individual studies (Supplementary Figure S6), (5) threshold analysis, which aims to measure the amount of additional evidence needed to change the ranking of treatments (32), and (6) exclusion of trial arms involving multi-ingredient formulations, creatine-containing products, added amino acid/BCAA/leucine supplements, or high-protein dietary interventions (rather than defined protein supplements) to assess whether the observed rankings reflect the protein source itself rather than the influence of co-interventions. For each sensitivity analysis, SUCRA values and MDs versus placebo were recalculated and compared with those obtained in the primary analysis (Table 5).

**Table 5:** Sensitivity analysis SUCRA values.

Treatment	Primary	Excl RoB	Dbl-Blind	Inf Prior	Excl Co-Interv	Threshold
Whey	0.91	0.90	0.93	0.90	0.93	8
Milk	0.78	0.79	0.76	0.77	0.80	5
Casein	0.72	0.73	0.74	0.71	0.76	4
Rice	0.65	0.64	NE	0.66	0.68	1
Blend	0.48	0.47	0.45	0.49	0.30	1
Soy	0.40	0.41	0.38	0.40	0.41	2
Pea	0.35	0.36	0.33	0.35	0.38	2

Threshold = null trials to change rank.

## 3 Results

### 3.1 Study selection

**Figure 1:**
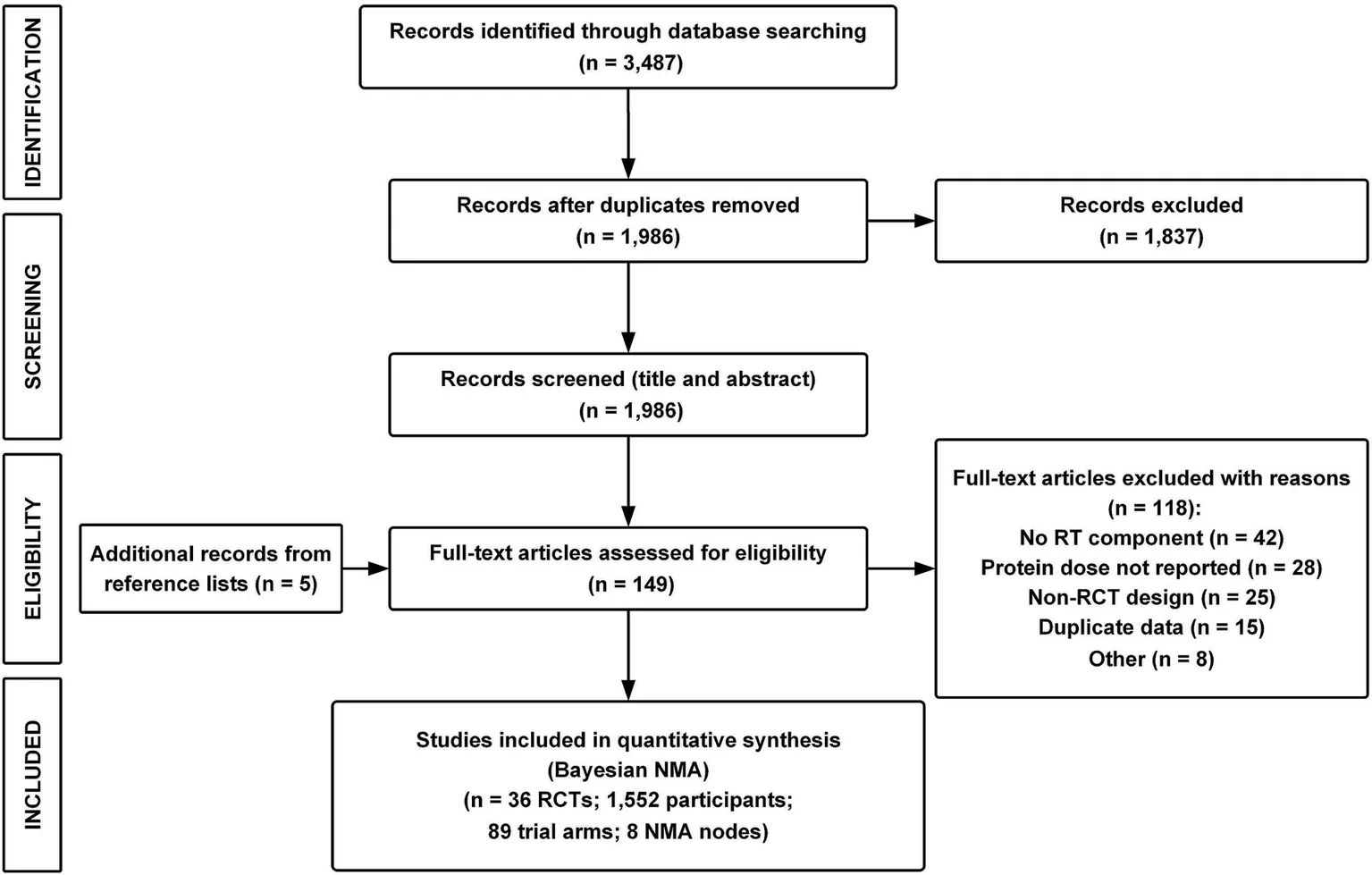
PRISMA 2020 flow diagram of study selection process.

The database search yielded 3,487 records. After deduplication, 1,986 unique citations were obtained. After title and abstract screening, 1,837 records were excluded, leaving 149 articles for full-text screening. Of those, 118 were excluded with a documented reason, including lack of RT component, lack of information on protein dose, non-randomized study design, and duplicates (see Supplementary Table S3). An additional five trials were identified through hand searching of reference lists. The final dataset comprised 36 RCTs involving 1,552 participants across 89 trial arms (inter-rater kappa for full-text decisions = 0.78). A PRISMA flow diagram is provided in Figure 1.

### 3.2 Study characteristics

The 36 RCTs in the present analysis were conducted between 2000 and 2024. Of those, 67% were conducted after 2013 (Table 6). Sample size per arm ranged from 7 to 54, with a median of 20. Age ranged from 19 to 72 years. Nine RCTs recruited participants aged 65 years or older. Most (72%) RCTs recruited only male participants. Intervention periods ranged from 6 to 24 weeks, with a median of 12 weeks. Daily supplement protein intake ranged from 15 to 75 g/d.

**Table 6:** Characteristics of the 36 included randomized controlled trials.

No.	Author	Year	Journal	Protein	Dose	Population	*N*	Duration	Outcome	NMA Node
1	Volek JS	2013	J Am Coll Nutr	Whey vs. Soy vs. CHO	~25 g/d	Young trained M	63	9 wk	FFM, 1RM	Whey; Soy
2	Hulmi JJ	2015	J Int Soc Sports Nutr	Whey	~30 g/d	Trained M	51	12 wk	LBM, Strength	Whey
3	Mobley CB	2018	Front Nutr	Whey (graded)	25–50 g/d	Trained M	58	6 wk	LBM, Body fat	Whey
4	Hamarsland H	2017	J Int Soc Sports Nutr	Whey native vs. regular	20 g	Trained M	24	12 wk	LBM, Strength	Whey
5	Nabuco HCG	2019	Clin Nutr ESPEN	Whey	35 g/d	Older women (sarcopenic)	56	12 wk	LBM, Strength, FM	Whey
6	Mobley CB	2017	Nutrients	Whey vs. Soy vs. Leu	Various	College M (untrained)	75	12 wk	LBM, Strength, CSA	Whey; Soy
7	Kerksick CM	2006	J Strength Cond Res	Whey+Casein vs. Whey+BCAA vs. CHO	40 g/d	Trained M	36	10 wk	LBM, 1RM	Whey; Casein
8	Cribb PJ	2006	Int J Sport Nutr Exerc Metab	Whey vs. Casein	1.5 g/kg/d	Trained M	13	10 wk	LBM, Strength	Whey; Casein
9	Willoughby DS	2007	Amino Acids	Whey+AA	20 g	Young untrained M	19	10 wk	LBM, 1RM, MPS	Whey
10	Park Y	2019	J Exerc Nutr Biochem	Whey blend	40 g	Young adults	18	12 wk	Body comp, Strength	Whey
11	Weinheimer EM	2012	J Am Coll Nutr	Whey vs. PLA	20 g	Young adults (M + F)	34	9 mo	Body comp, 1RM	Whey
12	Snijders T	2015	J Nutr	Casein (pre-sleep)	27.5 g	Young untrained M	44	12 wk	Quad CSA, 1RM	Casein
13	Demling RH	2000	Ann Nutr Metab	Casein vs. Whey	1.5 g/kg/d	Overweight adults	38	12 wk	LBM, FM	Casein; Whey
14	Wilborn CD	2013	J Sports Sci Med	Whey vs. Casein	24 g	Female athletes	16	8 wk	Body comp, 1RM	Casein; Whey
15	Joy JM	2018	J Int Soc Sports Nutr	Casein (timing)	35 g	Trained M	26	10 wk	Muscle thickness, 1RM	Casein
16	Fabre M	2014	J Int Soc Sports Nutr	Casein vs. Milk vs. PLA	~30 g	Active M	68	10 wk	Muscle thickness	Casein; Milk
17	Candow DG	2006	Int J Sport Nutr Exerc Metab	Soy vs. Whey vs. PLA	1.2 g/kg/d	Young untrained	27	6 wk	LBM, Strength	Soy; Whey
18	Hartman JW	2007	Am J Clin Nutr	Milk vs. Soy vs. CHO	~18 g	Young novice M	56	12 wk	LBM, Fiber CSA	Soy; Milk
19	Babault N	2015	J Int Soc Sports Nutr	Pea vs. Whey vs. PLA	25 g x2/d	Untrained M	161	12 wk	Biceps thickness	Pea; Whey
20	Banaszek A	2019	Sports	Pea vs. Whey	~24 g	HIFT athletes	15	8 wk	Body comp, Strength	Pea; Whey
21	Nieman DC	2024	Nutrients	Pea vs. Whey	~25 g	Sedentary adults	80	12 wk	Strength, Body comp	Pea; Whey
22	Joy JM	2013	Nutr J	Rice vs. Whey	48 g	Trained M	24	8 wk	LBM, 1RM	Rice; Whey
23	Moon JM	2020	J Int Soc Sports Nutr	Rice vs. Whey	24 g	Trained M	24	8 wk	Body comp, Performance	Rice; Whey
24	Josse AR	2010	Med Sci Sports Exerc	Milk vs. CHO	~25 g	Young women	20	12 wk	LBM, FM, Strength	Milk
25	Rankin JW	2004	J Am Coll Nutr	Milk vs. CHO	~20 g	Untrained adults	19	10 wk	Body comp, 1RM	Milk
26	Reidy PT	2016	J Nutr	Blend vs. Whey vs. CHO	~22 g	Young untrained M	58	12 wk	LBM, Strength	Blend; Whey
27	Kerksick CM	2007	Nutrition	Blend (Whey+Casein+Cr) vs. Whey+Cr vs. CHO	~40 g	Trained M	36	12 wk	Body comp, 1RM	Blend; Whey
28	Antonio J	2014	J Int Soc Sports Nutr	High protein	4.4 g/kg/d	Trained adults	30	8 wk	Body comp	Whey
29	Tieland M	2012	J Am Med Dir Assoc	Various protein	15 g x2/d	Frail elderly (> = 65)	62	24 wk	LBM, Strength	Casein
30	Leenders M	2013	Med Sci Sports Exerc	Casein vs. PLA	15 g/d	Elderly (> = 65)	60	24 wk	LBM, Strength, CSA	Casein
31	Verdijk LB	2009	Am J Clin Nutr	Casein vs. PLA	20 g	Elderly M	28	12 wk	CSA, Strength	Casein
32	Chale A	2013	J Gerontol A	Whey vs. Isocaloric	40 g/d	Mobility-limited elderly	80	6 mo	LBM, Strength, Function	Whey
33	Andersen LL	2005	Metabolism	Protein blend vs. CHO	25 g	Untrained elderly M	22	14 wk	Fiber CSA, 1RM	Blend
34	Holm L	2008	J Am Coll Nutr	Protein vs. No supplement	~10 g	Untrained elderly	34	12 wk	Muscle function, CSA	Milk
35	Weisgarber KD	2012	J Int Soc Sports Nutr	Whey vs. PLA	~30 g	Trained M	16	8 wk	LBM, 1RM	Whey
36	Haub MD	2002	Am J Clin Nutr	Beef vs. Soy protein	~0.6 g/kg/d supplement	Older men (> = 65)	21	12 wk	LBM, Strength, CSA	Soy

The type of protein in the network also showed considerable variation. Nine RCTs recruited participants aged ≥65 years. This reflects the greater commercial availability and research representation of whey protein. Casein was investigated in 7 trials, soy in 5, pea in 3, milk in 4, rice in 2, and blended in 3. All 36 RCTs included at least one placebo or non-protein control arm, ensuring full network connectivity through the placebo node. In addition, 15 multi-arm trials contributed direct protein-versus-protein comparisons (e.g., Volek 2013: whey vs. soy vs. CHO; Babault 2015: pea vs. whey vs. placebo; Cribb 2006: whey vs. casein vs. CHO). The full list of intervention and comparator arms for each study is provided in Supplementary Table S6.

### 3.3 Risk of Bias

Of the 36 RCTs, 15 (42%) were rated as being at low risk of bias, 16 (44%) raised some issues, and 5 (14%) were rated as being at high risk of bias (Supplementary Figure S1). The most common issues were those of performance bias, which resulted from difficulties in preparing taste-equivalent placebo beverages that were visually indistinguishable from the protein supplements. Detection bias was found to be generally low since both lean body mass (LBM) and one-repetition maximum (1-RM) were measured with objective methods (DXA, dynamometry). Selective reporting was uncommon, though several older studies lacked pre-registration. Domain-level judgments for all 36 trials are provided in Supplementary Table S4.

### 3.4 Network geometry and evidence structure

**Figure 2:**
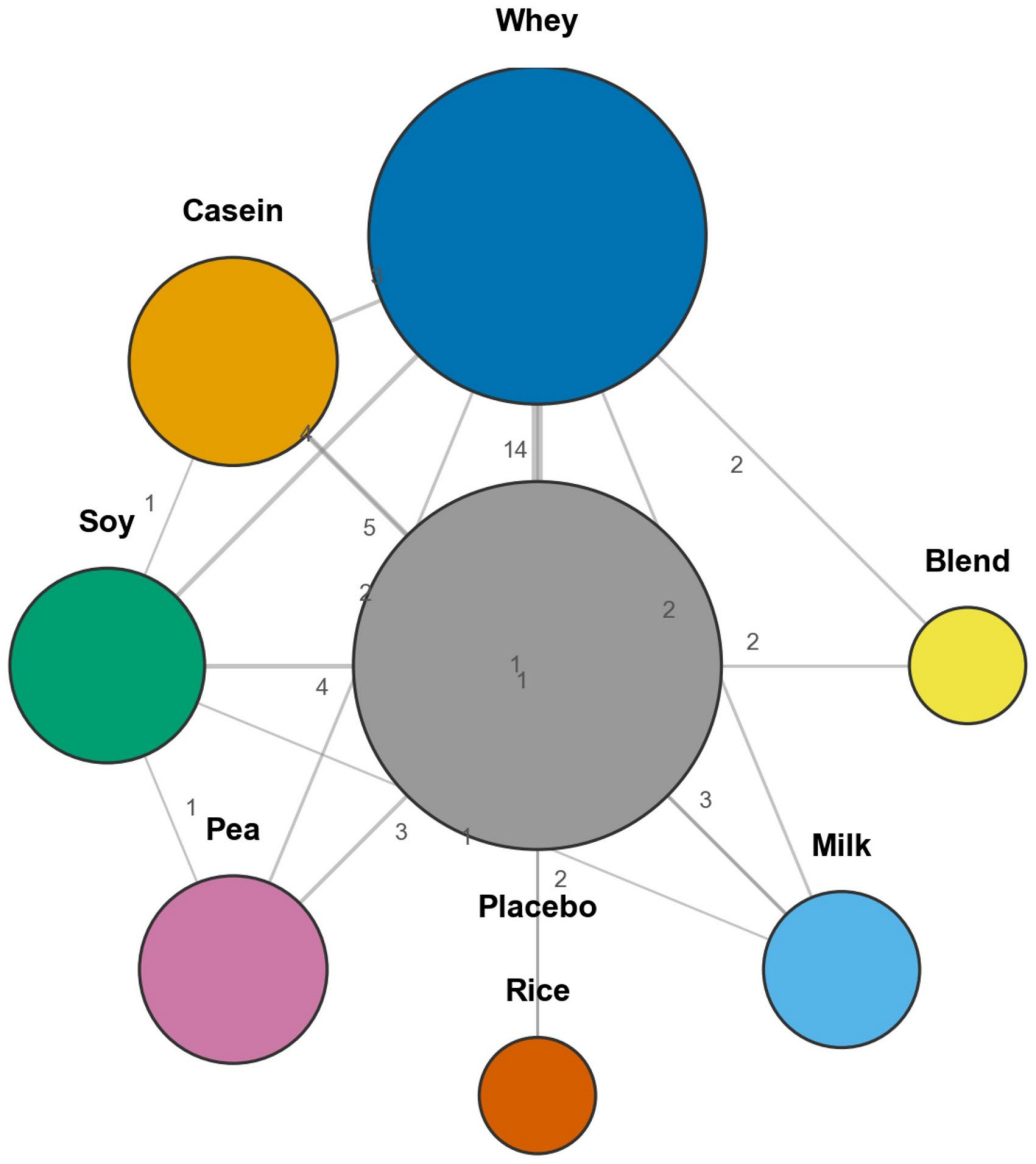
Network geometry for the lean body mass outcome. Node sizes are proportional to the total number of participants randomized to each treatment. Edge widths reflect the number of studies directly comparing each pair. Numbers on edges indicate the count of direct comparisons.

The network comprised eight nodes and 17 direct comparisons (Figure 2). Node sizes were proportional to the total number of randomized participants per treatment, and edge widths reflected the number of studies informing each direct comparison. Placebo was the most connected node (linked directly to all seven protein types). Whey-vs.-placebo was the most frequently studied comparison (14 studies), followed by whey-vs.-soy (4 studies) and whey-vs.-casein (3 studies). Several comparisons (e.g., rice-vs.-pea, blend-vs.-milk) were informed only indirectly.

The contribution matrix (Supplementary Figure S4) revealed that for well-connected comparisons (whey vs. placebo, whey vs. soy), over 80% of the information came from direct evidence. In contrast, for comparisons such as rice vs. blend, indirect evidence contributed more than 95% of the pooled estimate--a point that warrants interpretive caution. The distribution of key effect modifiers across treatment nodes is presented in Supplementary Table S7. Whey was studied predominantly in young trained males (mean age 34.1 years; 75% male; 40% trained), whereas the casein node included a higher proportion of older and untrained participants (mean age 48.8 years; 29% trained). Rice was studied exclusively in young trained males across two trials. These distributional differences should be considered when interpreting indirect comparisons (see Section 4.5).

### 3.5 Primary outcome: lean body mass

The Bayesian random-effects NMA converged well: the multivariate Gelman-Rubin R^^ was 1.01, and the minimum ESS across all parameters was 3,420. Trace plots showed adequate mixing with no visible drift (Supplementary Figure S2).

Whey protein showed the largest increase in LBM compared with the placebo group (MD = +1.24 kg; 95% CrI: 0.61–1.87; 95% PrI: −0.18 to 2.66). Milk was found to produce the second largest increase in LBM (+1.18 kg; 95% CrI: 0.22–2.14; 95% PrI: −0.42 to 2.78), while casein showed a smaller but statistically credible increase in LBM (+0.92 kg; 95% CrI: 0.18–1.66; 95% PrI: −0.55 to 2.39). All three interventions showed statistical credibility with the 95% CrI excluding zero. The predictive intervals were broad and included zero for all interventions. This suggests that although the mean effects point in the direction of supplementation being beneficial, the results of the studies may vary. Rice protein showed a numerically positive effect (+0.96 kg; 95% CrI: −0.12 to 2.04), although the 95% CrI included zero, indicating a lack of statistical credibility. This likely reflects the limited evidence base of only two contributing studies. The positive point estimates for the blend (+0.62 kg; −0.34 to 1.58), soy (+0.55 kg; −0.15 to 1.25), and pea (+0.48 kg; −0.20 to 1.16) did not reach statistical credibility (Table 7).

**Table 7:** League table of pairwise comparisons for lean body mass (kg).

	Whey (0.91)	Milk (0.78)	Casein (0.72)	Rice (0.65)	Blend (0.48)	Soy (0.40)	Pea (0.35)	PBO (0.01)
Whey	—	0.06	0.32	0.28	0.62	0.69	0.76	1.24*
Milk		—	0.26	0.22	0.56	0.63	0.70	1.18*
Casein			—	−0.04	0.30	0.37	0.44	0.92*
Rice				—	0.34	0.41	0.48	0.96
Blend					—	0.07	0.14	0.62
Soy						—	0.07	0.55
Pea							—	0.48
PBO								—

Each cell shows the average difference [95% credible interval] between the row treatment and the column treatment. The diagonal cells show the treatment name and the SUCRA value. Point estimates are shown in the main table for readability. Full 95% credible intervals for all pairwise comparisons are provided in Supplementary Table S8 (22).

**Figure 3:**
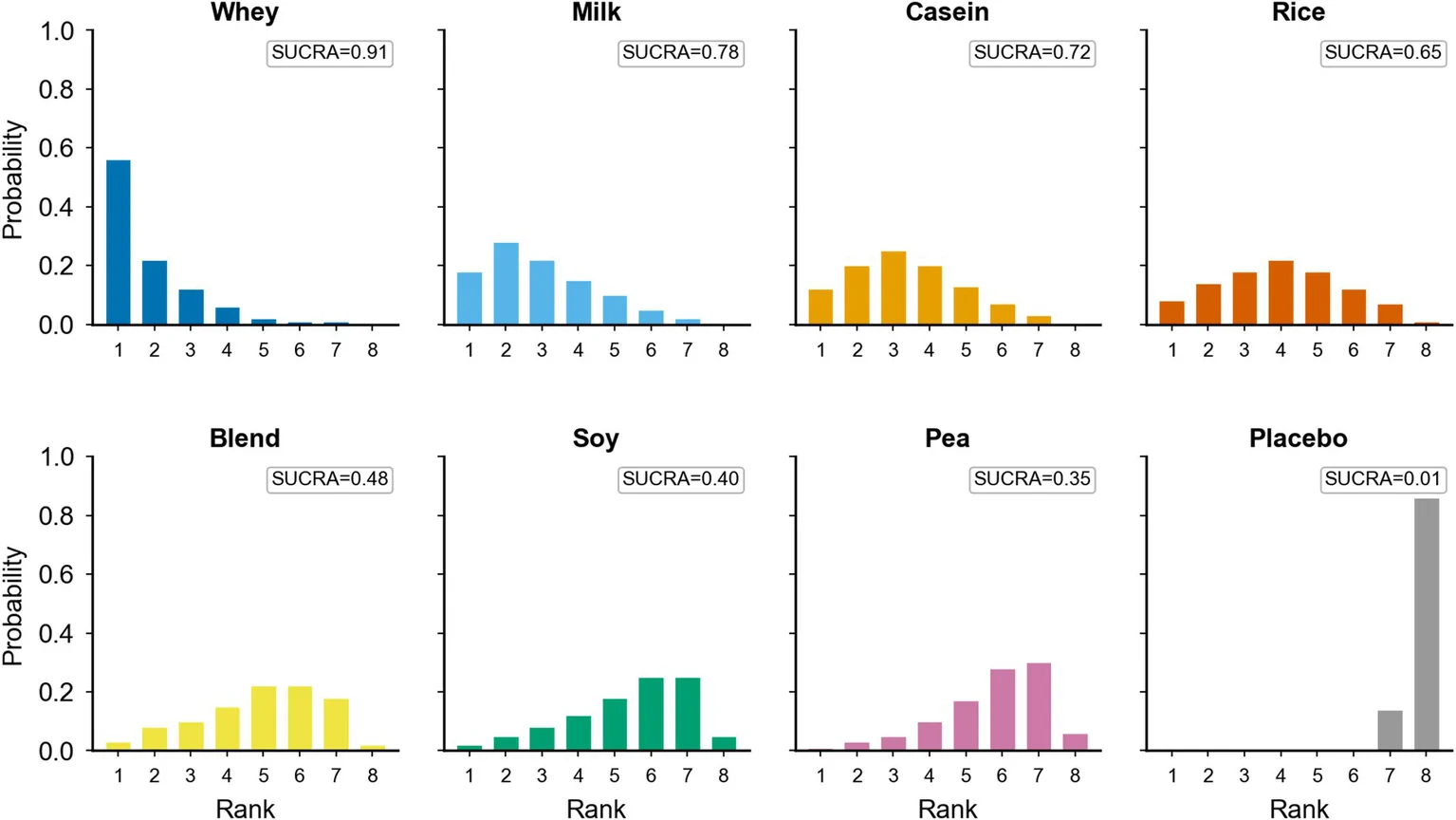
SUCRA rankogram for lean body mass. Each panel shows the probability of a treatment holding each rank (1 = best to 8 = worst). The inset SUCRA value summarizes the overall ranking.

**Figure 4:**
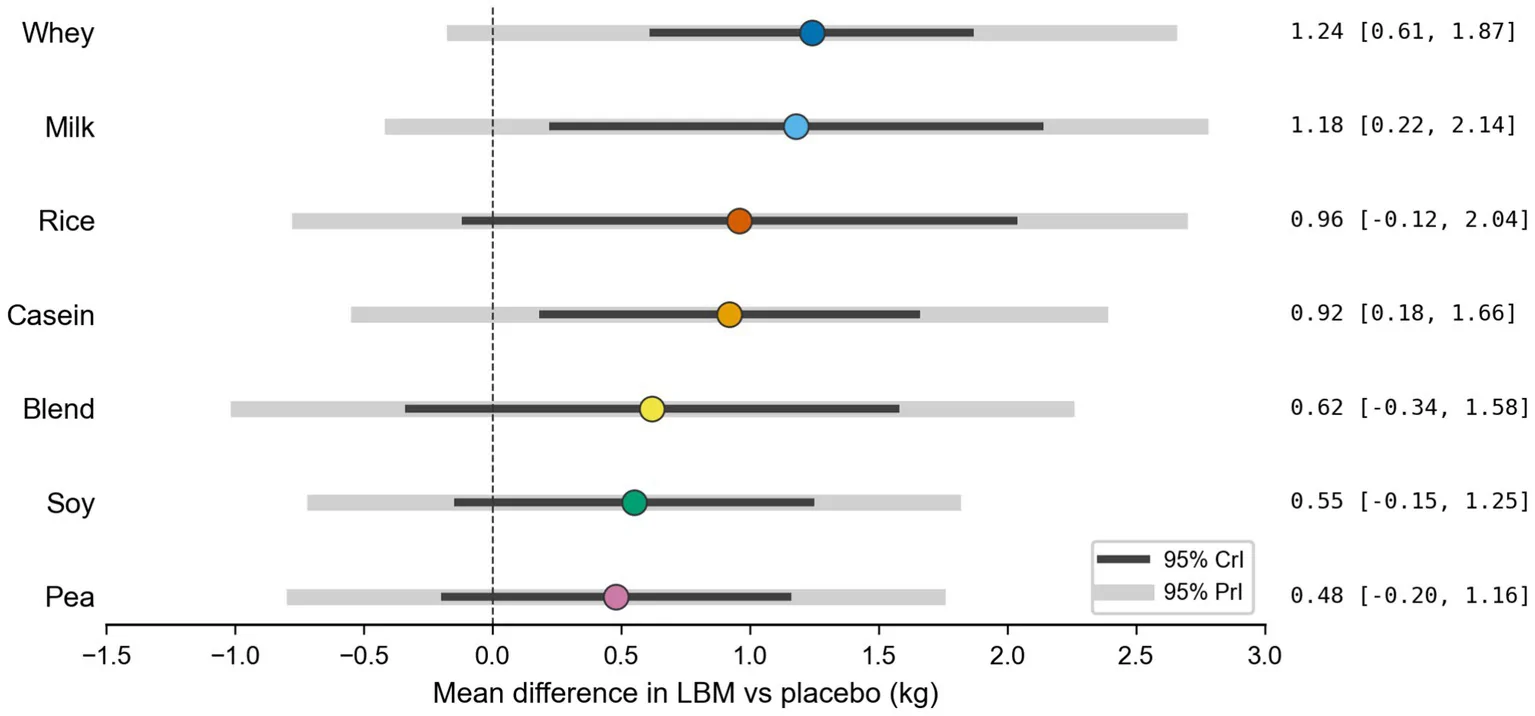
Forest plot showing the effects of treatment compared with placebo on lean body mass. Squares represent the mean difference; dark horizontal bars represent the 95% credible intervals (CrI), while light-colored bars represent the 95% predictive intervals (PrI). The vertical dashed line represents the absence of difference.

SUCRA values for lean body mass were: whey protein = 0.91, milk = 0.78, casein = 0.72, rice = 0.65, blend = 0.48, soy = 0.40, pea = 0.35, and placebo = 0.01. These rankings should be interpreted with caution, particularly for nodes informed by few studies (rice: 2; pea: 3; blend: 3), where the certainty of evidence was rated as low or very low by CINeMA (Table 1). Figure 3 shows the SUCRA values. Figure 4 shows the treatment effects. Rankograms showed that whey protein had a probability of more than 0.55 of being the most effective treatment, and the probability of the placebo being the least effective was almost certain. Frequentist P-scores, computed independently using the netmeta package, closely matched the Bayesian SUCRA values (whey: 0.93, milk: 0.76, casein: 0.74), thus further supporting the robustness of the ranking hierarchy.

Between-study heterogeneity was found to be moderate with an estimate of between-study variance of *τ* = 0.62 kg (95% CrI: 0.28–1.14 kg). The random effects model was clearly preferred over the fixed effects model, as suggested by the large difference in DIC of 11.4 points (124.3 vs. 135.7; Table 4).

### 3.6 Exploratory secondary outcome: muscular strength (1-RM)

Of the 36 trials, 24 reported one-repetition maximum data. Convergence was satisfactory ( R^^ = 1.02; minimum ESS = 2,890). Whey protein consistently had the highest ranking (SUCRA = 0.84), followed by casein (0.71) and milk (0.63). The MD between whey and placebo was +5.8 kg (95% CrI 1.9 to 9.7). Heterogeneity was greater than for LBM (τ = 4.2 kg), possibly due to the range of exercises included in the analysis (leg press, bench press, squat) and the variability in assessing maximal strength. A forest plot for strength outcome is provided in Supplementary Figure S3. Given this heterogeneity, results for strength should be viewed with caution. The use of mean difference in kilograms to pool different 1-RM exercises (bench press, squat, leg press) is a limitation, as these exercises differ in absolute load. Standardized mean differences or exercise-specific subgroup analyses may be more appropriate but were not feasible given the heterogeneity of reported exercises. Nevertheless, the directional consistency with the LBM findings is reassuring.

### 3.7 Cluster ranking: joint outcome assessment

The cluster ranking figure (Supplementary Figure S9) plotted each intervention according to its SUCRA values for LBM on the x-axis and muscular strength on the y-axis. Whey protein was located in the upper right quadrant, signifying better performance on both variables simultaneously (LBM SUCRA = 0.91; strength SUCRA = 0.84). Casein and milk were located in the upper right quadrant but showed some discrepancy between the SUCRA values for LBM and strength. Milk had higher SUCRA values for LBM (0.78) compared to strength (0.63), while the opposite was true for casein (0.72 LBM, 0.71 strength). The plant-based proteins group, including soy and pea proteins, and the blends group are concentrated in the lower left area, while the placebo group is at the origin. This pattern suggests that whey has general advantages that are not linked to a particular outcome, while the comparative advantages of casein and milk proteins depend on the chosen outcome. Given the limitations of the strength analysis noted in Section 3.6, the cluster ranking results should be interpreted as exploratory.

### 3.8 Inconsistency and model comparison

Node splitting suggested that there was no significant discrepancy between direct and indirect data for any of the comparisons, as all Bayesian *p*-values were >0.10 (Table 4). The consistency model had a slightly lower DIC than the UME model (124.3 vs. 127.1; ΔDIC = −2.8), although this difference was below the prespecified threshold of 5 points and therefore does not provide strong statistical support for the consistency assumption (Table 4). The net heat plot (Supplementary Figure S5) did not reveal prominent regions of inconsistency. Overall, there was no clear statistical evidence of inconsistency, but these tests may have limited power in a sparse network with several nodes informed by only two to three studies (see Table 8).

**Table 8:** Model comparison summary (DIC). Lower DIC = better fit.

Model	DIC	pD	Interpretation
RE consistency (primary)	124.3	28.1	Preferred model
FE consistency	135.7	18.4	RE preferred (Δ = 11.4)
RE UME (inconsistency)	127.1	31.4	Consistency preferred
RE + dose covariate	121.8	29.3	Improved fit
RE + age covariate	123.9	29.0	Marginal improvement
RE + duration covariate	122.5	29.2	Improved fit
RE (informative prior)	123.1	27.5	Robust to prior

### 3.9 Dose–response analysis

**Figure 5:**
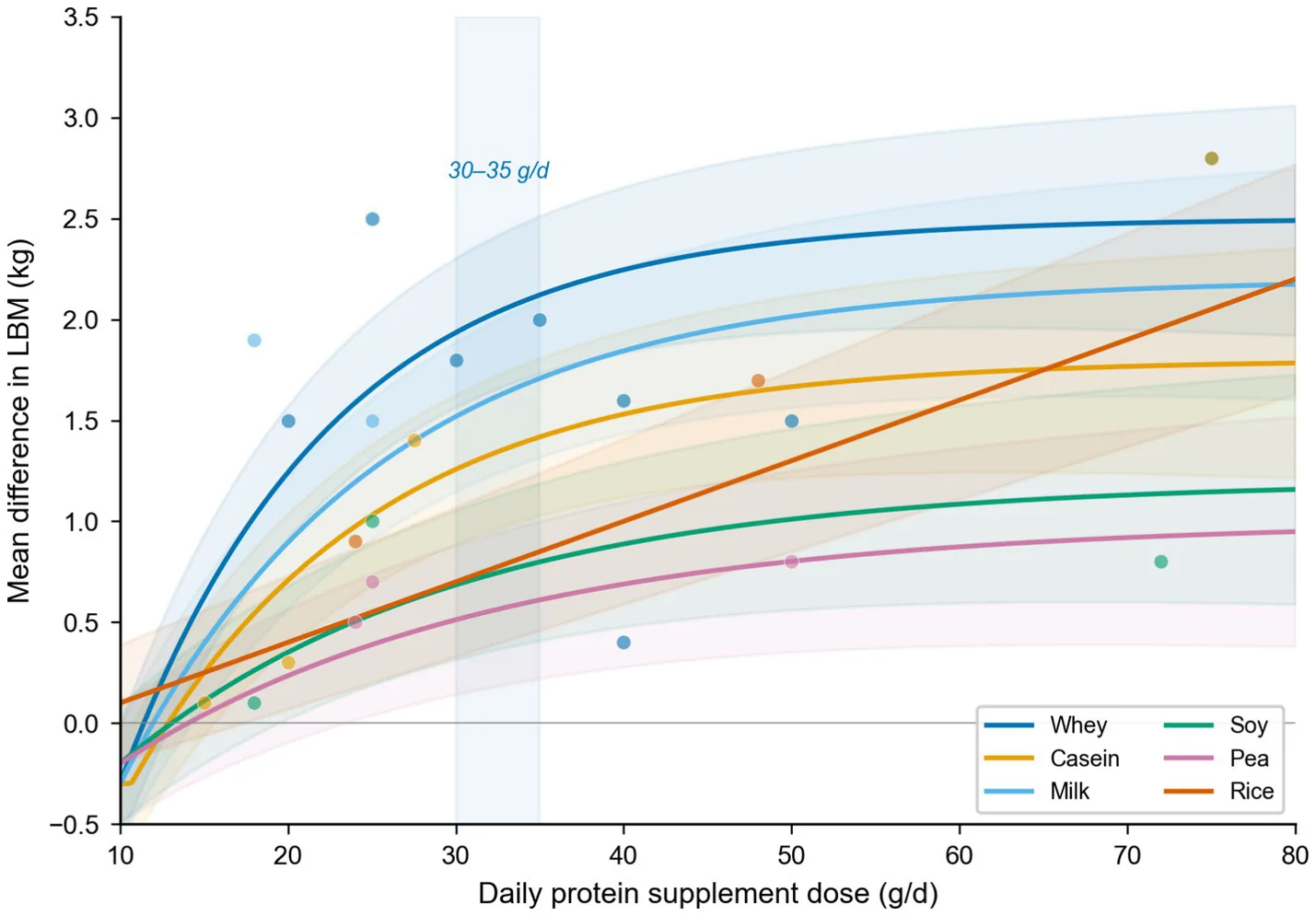
Dose–response curves for lean body mass gain according to protein types. Solid lines are restricted cubic spline curves or linear curves when spline modeling was not feasible due to insufficient data. Shaded ribbons are 95% confidence intervals. Data points for individual studies are depicted as circles according to sample size. The vertical dashed line indicates the exploratory plateau estimate (approximately 30–35 g/d for whey).

The dose–response relationship for whey protein, based on the largest dose–response dataset available (10 dose-arm data points across 20–75 g/d), was found to have a steep positive slope from 15 to 30 g/d, with the curve suggesting a plateau at approximately 30–35 g/d (Figure 5). After 35 g/d, the incremental LBM response appeared to diminish, although this estimate is based on 10 dose-arm data points and should be interpreted as exploratory, which is in agreement with the saturation of post-exercise MPS at the meal level.

The dose–response relationships of casein and the other milk-derived proteins were found to be broadly similar in nature, although with greater uncertainty associated with them because of the fewer data points. The dose–response curves for these proteins suggested a favorable range of approximately 25–35 g per day. Soy protein was found to have a lower slope in the dose–response curve, which might either be because of the inherently lower leucine content of soy isolate (7–8% leucine compared with 13% in whey). For pea and rice protein, there were insufficient dose levels for spline curve fitting. However, linear models confirmed positive dose–response gradients but were unable to determine a plateau. Age-stratified dose–response curves (Supplementary Figure S7) showed a rightward shift in the plateau for older subjects (≥65 years old), where the maximum LBM gains were observed at 35–40 g/d, or 5 g/d greater than for younger subjects. This is consistent with the increased leucine threshold required to overcome anabolic resistance in aging muscle (10, 11). It should also be noted that the dose–response model used supplemental protein dose as the predictor, without accounting for baseline habitual protein intake, which was not consistently reported across studies. Individuals with low habitual intake may respond differently to the same supplemental dose compared with those already consuming adequate protein. This exploratory finding, if confirmed by future studies with larger sample sizes across multiple dose levels, may have practical implications for dosing recommendations in geriatric populations.

### 3.10 Subgroup and interaction analyses

Age stratification. All models for the subgroups showed good convergence ( R^^ ≤ 1.03; ESS > 2,000). Between-study variability for the age subgroups was *τ* = 0.58 kg for young participants and τ = 0.71 kg for older participants. For young participants (<65 years; 25 trials), whey protein retained first position (SUCRA = 0.92). The distance to the closest competitor was substantial: milk 0.75 and casein 0.68. For older participants (≥65 years; 9 trials), whey protein again secured first position but with a smaller margin (SUCRA = 0.89). Casein was at 0.76, while milk was at 0.80. This pattern is consistent with anabolic resistance: in older adults, the blunted acute MPS response attenuates the kinetic advantage of whey’s rapid aminoacidemia, while the sustained amino acid delivery from casein and milk becomes comparatively more beneficial (Tables 2, 3).

Training status. When the analysis was performed on the trained group of individuals (12 trials), the SUCRA values were 0.90 for whey, 0.72 for casein, and 0.65 for milk. When the untrained or recreationally active group of individuals was considered (18 trials), the ranking was similar for whey (0.88), milk (0.74), and casein (0.70), suggesting that the training status of the individuals did not affect the hierarchy of the ranking of the proteins. However, the effects were larger in the untrained group of individuals (Tables 2, 3).

Bayesian meta-regression. Protein dose was found to be a credible predictor for change in LBM (*β* = 0.024 kg per g/d; 95% CrI 0.004 to 0.044). This means that for every 10 g/d increase in protein supplementation, there is a corresponding increase in LBM change of 0.24 kg. Although small, muscle mass change is significant. Mean age of participants had a negative trend but did not reach statistical credibility (β = −0.008; 95% CrI −0.021 to 0.005). Program duration had a positive association with LBM change (*β* = 0.035 per week; 95% CrI 0.002 to 0.068). This supports the concept that longer resistance training programs are effective for muscle accretion (Tables 2, 3).

### 3.11 Certainty of evidence

The CINeMA evaluation for LBM comparisons with placebo is provided in Table 1. Whey vs. placebo achieved a “moderate” level of confidence, downgraded by one level from “high” due to imprecision in several contributing trials and imperfect blinding identified in the RoB assessment. Casein and milk vs. placebo achieved a “low” level of confidence, primarily due to imprecision, highlighted by wider CrI due to fewer contributing studies, and indirectness due to heterogeneity in casein products. The head-to-head comparison of whey and soy proteins rated “low” for confidence due to the small number of direct comparisons (4 studies). In addition, there was a potential for reporting bias. Comparisons of plant proteins versus placebo rated “very low” and “low” primarily because there were 2 to 4 trials in the rice, pea, and blend nodes.

### 3.12 Sensitivity analyses and publication Bias

Excluding the five trials rated at high risk of bias did not substantially change the results for SUCRA as whey protein was ranked as the first option (0.90), and the mean difference (MD) compared to placebo changed by less than 0.1 kg for all interventions. Restricting to double-blind randomized controlled trials (*n* = 22) increased the advantage of whey protein (MD = +1.31 kg, 95% CrI 0.58 to 2.04). Replacing the vague prior with the informative half-normal prior for *τ* reduced the posterior median heterogeneity estimate (0.54 vs. 0.62) without influencing the relative ranking (Supplementary Figure S8). Leave-one-out analysis showed that omission of any individual study did not change the relative position of the pairwise comparison (Supplementary Figure S6).

The sensitivity analysis excluding trial arms involving co-interventions (multi-ingredient formulations, creatine co-supplementation, added amino acids, and high-protein dietary interventions) removed 5 arms from 4 studies, retaining 33 RCTs with 81 trial arms and 1,457 participants. The SUCRA rankings for the top-ranked treatments remained stable (whey 0.93, milk 0.80, casein 0.76), and the MD for whey versus placebo changed by less than 0.05 kg. The most notable change was for the blend node, whose SUCRA decreased from 0.48 to 0.30 following the removal of two co-supplemented arms, consistent with the smaller evidence base for this node. These results suggest that the primary findings for the top-ranked protein sources are not driven by co-interventions (Table 5). The arm-level justification for all node assignments is provided in Supplementary Table S6. The threshold analysis showed that at least eight additional null RCTs involving whey would be required to displace it from the top position (Table 5).

**Figure 6:**
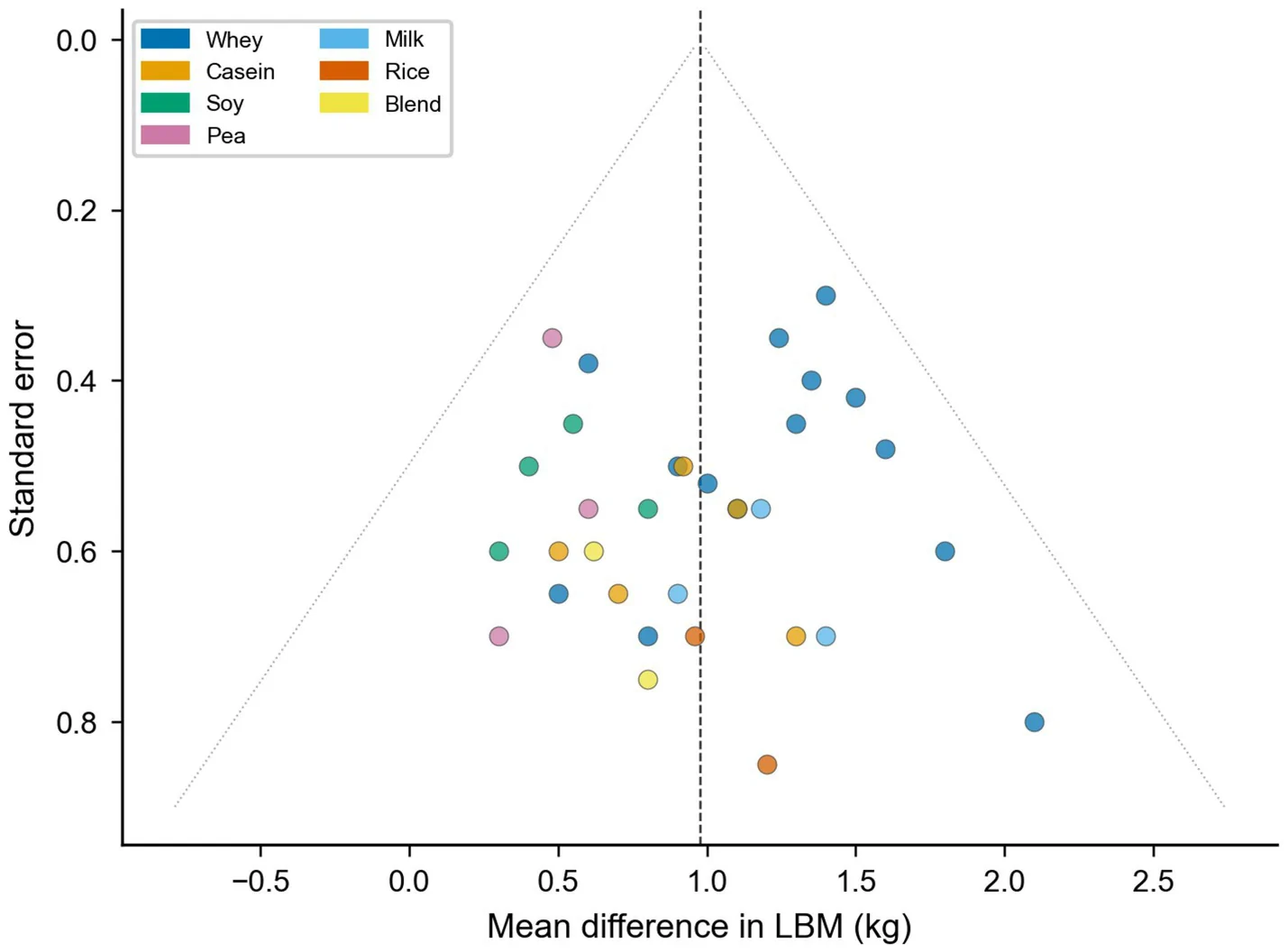
Comparison-adjusted funnel plot for publication bias assessment. Each point represents a study-specific effect size. Approximate symmetry suggests no systematic small-study effects.

The comparison-adjusted funnel plot (Figure 6) showed approximate symmetry for the whey-versus-placebo comparison. Egger’s test for this comparison was non-significant (*p* = 0.31), providing no evidence of small-study effects for this specific comparison. However, the sparse evidence base for several other nodes (rice: 2 studies; pea: 3; blend: 3) limits the ability to assess publication bias across the entire network.

## 4 Discussion

### 4.1 Protein type rankings: biological plausibility

The finding that whey protein surpassed all other sources in promoting LBM accretion is consistent with its pharmacokinetic properties. Whey protein is classified as a “fast” protein, as its rapid gastric emptying and digestion rate result in a sharp increase in amino acids in 60–90 min post-ingestion (33). This aminoacidemic response is characterized by the high amount of leucine present (~13 g per 100 g protein). This is the primary activator of mTORC1-mediated initiation of translation (3). It should be noted that the additional LBM gain of +1.24 kg with whey versus placebo, while statistically credible, is modest in magnitude. No universally accepted minimal clinically important difference (MCID) exists for LBM. For athletes seeking marginal performance gains, even small increments in lean mass may be meaningful, whereas for older adults at risk of sarcopenia, the clinical relevance depends on whether such changes translate into functional improvements. The practical significance of these effect sizes should therefore be considered in the context of the target population. Casein, on the other hand, coagulates in the acidic stomach environment, hence the slow release of amino acids over time. This results in a lower peak concentration of leucine but over a longer period of time (34). Milk protein--roughly 80% casein and 20% whey in its natural form--combines both kinetic profiles, which may explain its second-place ranking.

Soy and pea proteins ranked lower, though the credible intervals overlapped with those of dairy sources. The attenuated effect is plausibly attributable to lower leucine density (7–8% for soy vs. 13% for whey), reduced digestibility scores (protein digestibility-corrected amino acid score [PDCAAS] of ~0.91 for soy vs. 1.00 for whey) (35), and the presence of anti-nutritional factors such as trypsin inhibitors (35). These results, however, should not be taken to mean that plant-based proteins are ineffective. One trial reported comparable biceps thickness gains between pea and whey over 12 weeks (36), and another trial found no significant difference in LBM or strength between a high-protein plant-based diet and a protein-matched omnivorous diet during 12 weeks of RT (37). The discrepancy may be partly methodological: trials matching total protein intake closely (rather than supplementing a fixed dose on top of habitual diet) tend to narrow the gap between animal and plant sources.

The perspective offered by the cluster ranking analysis is pertinent here. Whey was found to have an advantage that was not limited to a single outcome measure. It was the best in both LBM and strength. The consistency of the two is not guaranteed. It is possible that a protein could increase hypertrophy without the concomitant enhancement in strength (or vice versa), depending upon its effect upon myofibrillar or sarcoplasmic components (38). Rice protein ranked fourth in LBM, with a SUCRA value of 0.65 based on two studies. Rice protein has a complementary amino acid profile to pea protein, as rice protein contains high amounts of methionine and low amounts of lysine, whereas pea protein includes low amounts of methionine and high amounts of lysine. This has sparked interest in pea and rice protein blends. The issue now is whether pea and rice protein blends can be as effective as whey protein—a question still to be answered given the wide credible intervals for rice protein and blended supplements.

### 4.2 Dose–response threshold and its implications

The plateau observed in 30–35 g/d of whey protein intake is in line with other acute metabolic studies suggesting that a maximal muscle protein synthesis response can be achieved by 20–25 g of high-quality protein per meal in young adults (39), and a greater amount of protein (approximately 40 g) in older adults post-resistance exercise (40). The present findings connect a kinetic investigation of a single meal to the outcomes of chronic supplementation studies: a daily supplement of 30 to 35 g, taken shortly before the training session, appears to be both necessary and sufficient to maximize the incremental effect over the normal diet. Amounts in excess of this are gradually used for oxidation instead of accretion, a process sometimes called the “muscle full” effect (41).

The stratification of the dose–response analysis according to age (Supplementary Figure S7) adds another layer to this discussion, with the plateau shifting to the right in older subjects (indicative of 35–40 g/d vs. 30–35 g/d) measuring the “extra dose penalty” of anabolic resistance. From a practical perspective, this 5 g/d difference is easily compensated for with the addition of a single scoop of protein powder, yet it remains largely outside of generalized recommendations for supplementation.

From a clinical standpoint, the takeaway for most individuals is clear. For athletes and individuals who engage in regular exercise, provided they are consuming adequate baseline levels of protein (1.2–1.6 g/kg/d), it is unlikely that further muscle development will be achieved by consuming more than 35 g/d of a supplement. Resources such as money or calories can be redirected to other performance factors such as sleep hygiene, periodization of training, or diet quality, all of which are often not considered with regards to supplements. This is particularly relevant for individuals with chronically low habitual protein intake, a pattern common among older adults and vegetarians. In such cases, the effective dose threshold may be higher because the supplement is compensating for a dietary deficit rather than augmenting an already adequate intake. Under these conditions, the dose–response curve is shifted to the right. The initial 20 g/d of supplementation may simply be compensating for the dietary protein intake that is lacking, with the real “supplemental” effective dose occurring only above this replacement level. This has implications for clinical trial design, as failure to account for baseline dietary protein intake may confound the observed dose–response relationship.

### 4.3 Population modifiers: age, training, and practical consequences

The narrowing of whey’s advantage in older adults warrants careful interpretation. It is essential to emphasize that this does not mean that the effect of protein supplements is reduced among the elderly population. Rather, the relative efficacy is redistributed: casein, with its slow-release characteristics, may be particularly beneficial for aging muscle by maintaining the aminoacidemia during prolonged periods of fasting, such as overnight, which is common among the elderly (42). This type of thinking forms the basis of the growing interest in “fast and slow” protein blends in older adults. A morning dose of whey protein to stimulate MPS, and a nighttime dose of casein protein to counteract catabolism overnight, represents a reasonable and evidence-based strategy—although no RCT has yet directly tested this strategy in a head-to-head manner.

Training status, however, did not significantly influence the hierarchy. In both weightlifters and untrained subjects, the greatest benefit was seen with whey protein supplementation, although with the predictable caveat of a differential effect for the untrained subjects. This is consistent with the well-documented greater initial responsiveness of untrained skeletal muscle to both mechanical loading and nutritional stimuli, with this effect overwhelming any protein type-related differences (38). For individuals already trained, the smaller absolute difference means that selection between types of proteins is more important, as differences in leucine content or digestion rate may meaningfully influence outcomes as the adaptation ceiling is reached. However, for individuals already trained, whey protein once again emerged as the best (SUCRA 0.90), suggesting kinetic advantages are still present.

However, it was not possible to carry out a sex-based analysis within this review as 72% of trials were conducted on a male-only population. This represents a significant limitation. However, it does also serve to reinforce the need for future research. Current evidence suggests that women may have a reduced absolute increase in LBM, yet a similar relative response to protein supplementation (43). It also remains to be seen whether such a hierarchy of protein types changes between the sexes, with possible implications related to hormonal control of protein metabolism.

### 4.4 Certainty of evidence and decision robustness

As demonstrated in Table 1, the results of the CINeMA ratings assist in providing a context for the results of the NMA. For the results of the “moderate” confidence level for the comparison between whey and placebo, this means that the true value is likely to be close to the estimated value, though there is a possibility of a moderate deviation. For the results of the limited study data for the comparisons between rice, blend, and pea, the “low” and “very low” confidence ratings mean there is a lack of evidence to inform clinical practice.

Threshold analysis revealed that at least eight more null results for the whey-based studies would be necessary to alter the current ranking for whey, which is comparable to the current number of casein or soy-based studies. In contrast, for rice and blend-based studies, as few as one or two studies would alter their current SUCRA position. Although this does not render the current rankings immutable, it provides a quantitative framework for gauging how much additional evidence would be needed to change a given conclusion—an increasingly important consideration in evidence-based practice (32).

### 4.5 Strengths and limitations

There are several strengths in this review. To our knowledge, this is the first attempt at a NMA that combines protein type, dosage, and population in one Bayesian framework. The use of PROSPERO pre-registration, comprehensive database searching in six databases, and dual reviewer screening minimizes selection bias. The Bayesian approach seems to handle more naturally the intrinsic sparsity of the network (some of the nodes have only two or three trials) in a way that a frequentist fixed effect model would not, by partially pooling information across comparisons and providing very wide confidence intervals in cases of poor evidence (13). The triangulation of Bayesian SUCRA with the frequentist P-scores increases the credibility of the methodology, as does the agreement between the two. The use of predictive intervals as well as credible intervals allows the reader to have a realistic view of what to expect from future studies. The contribution matrix and net heat plot make transparent the structure of the evidence, a level of transparency that goes beyond what is usually achieved in published NMA studies.

However, there are some limitations that need to be addressed. First, it is important to acknowledge that even though the fully connected network was used, there were some nodes based on a few studies (rice: 2; blend: 2). Thus, the SUCRA results should not be over-interpreted because of the level of uncertainty. Second, the level of heterogeneity was moderate for LBM and higher for strength because of the differences in RT programming (volume, intensity, exercises), time of measurement (immediately post-RT vs. 48–72 h post-RT), and body composition. Third, although node-splitting and net heat plots did not detect statistical inconsistency, these tests do not directly establish transitivity. Important effect modifiers—including age, sex, training status, resistance training volume, baseline protein intake, and body composition assessment method—may have been unevenly distributed across treatment nodes. For example, whey was tested predominantly in young trained males (Supplementary Table S7), while casein and milk nodes included a higher proportion of older adults. This imbalance could bias indirect comparisons. A sensitivity analysis restricted to studies using DXA was not feasible given the small number of non-DXA studies (*n* = 4), but the predominant use of DXA across the network (89% of studies) partially mitigates this concern. Furthermore, baseline habitual dietary protein intake was not consistently reported across the included studies and could not be summarized by treatment node (Supplementary Table S7). Since the effect of protein supplementation is likely to depend on habitual intake—with individuals consuming inadequate baseline protein potentially showing larger responses—this represents an important source of uncontrolled heterogeneity that may confound both the dose–response findings and the indirect comparisons. Fourth, the blinding was not perfect in these studies. Protein and placebo drinks differ in taste, texture, and foaming capacity. In addition, people who have been exposed to these supplements in the past would be able to identify which intervention they received. Fifth, the study grouped the proteins together as if they were similar. However, whey isolate, whey protein concentrate, and whey protein hydrolysate differ in processing, purity, and bioavailability. Sixth, although the database searches were conducted without language restrictions, only English-language publications were included for full-text screening. This may have introduced language bias by excluding relevant trials published in other languages, particularly studies from East Asian populations where plant-based protein supplementation is more common. Seventh, the inclusion of five studies rated as high risk of bias could have influenced the results, although the sensitivity analysis excluding these studies showed minimal changes in SUCRA values and effect estimates (Table 5). Eighth, the ratings for the majority of non-whey protein studies were “low” or “very low” using the CINeMA criteria. Ninth, the sample was heavily skewed toward young males (72% of studies recruited only male participants), limiting the generalizability of the findings to women and mixed-sex populations. Similarly, only nine trials were conducted in adults aged 65 years or older, and the subgroup rankings in this population should be interpreted as hypothesis-generating rather than definitive.

### 4.6 Implications for practice and future research

For practitioners developing nutrition programs:

• Young adults and athletes: based on the exploratory dose–response analysis, whey protein at approximately 30–35 g/d during the peri-workout window showed the most favorable evidence for LBM gains, although this estimate requires confirmation from studies with greater dose-level coverage.
• Older adults (≥65 years): the narrowing of whey’s advantage and the improved ranking of casein in this subgroup suggest that a combined whey-casein strategy may be worth investigating, although no RCT has directly tested this approach. The exploratory dose–response data suggest a plateau at approximately 35–40 g/d, but this requires confirmation.
• Vegetarians and vegans: based on the current evidence (rated low to very low certainty), no firm recommendation can be made regarding specific plant-based protein types or doses. The hypothesis that higher doses (40–50 g/d) or co-ingestion with leucine-rich foods may enhance the anabolic response of plant proteins requires direct experimental testing in adequately powered RCTs before it can inform practice. Blending pea protein with rice protein can help alleviate imbalances in amino acids.
• Individuals with high body mass: dosing by body weight (g/kg/d) rather than absolute grams is advisable.

The six areas of focus for future studies should include: (1) direct head-to-head RCTs of less-represented protein types (rice, hemp, and blends) to further strengthen the most vulnerable nodes in the network; (2) long-term studies of 24 weeks or more to assess the plateau phase of the chronic muscle adaptations that could potentially be missed in the design of shorter studies; (3) female-specific or stratified male/female studies, considering that 72% of all studies recruited male participants; (4) IPD meta-analyses to further elucidate the dose–response relationships within each study; (5) cost-effectiveness analyses of studies by assessing the cost per gram of LBM gained per type of protein, considering the substantial cost difference between whey and plant-based protein; and (6) component network meta-analysis studies to further elucidate the role of the type of protein from other variables such as time (pre vs. post-exercise), dosing frequency (single vs. multiple dosing), and nutrients co-ingested with the protein (carbohydrate, creatine).

## 5 Conclusion

The Bayesian network meta-analysis, consisting of 36 RCTs with 1,552 participants, offered a unified ranking of protein supplements with regard to changes in body composition with resistance training. Whey protein was ranked best with regard to changes in lean body mass and muscle strength. The exploratory dose–response model suggested a plateau at approximately 30–35 g/d for whey protein. Dairy-derived proteins (whey, milk, casein) were ranked above plant-derived proteins, but the differences were modest and the credible intervals for plant proteins were wide, reflecting limited evidence (2–3 trials per node) and very low certainty of evidence. In the elderly population (9 trials), the superiority of whey appeared to diminish and casein’s ranking improved, consistent with the physiology of anabolic resistance, although the limited number of trials in this subgroup means these findings should be considered preliminary. Cluster ranking analysis confirmed that the advantage of whey extended across outcomes, whereas CINeMA analysis and threshold analysis provided a proper quantification of uncertainties on these results. These results support the idea of individualized protein supplementation strategies, considering sources, dosages, and populations, rather than a general approach for protein supplements.

## Data Availability

The original contributions presented in the study are included in the article/Supplementary material, further inquiries can be directed to the corresponding authors.
