# Supplementary material for: Dose–structure–population interactions of protein supplementation combined with resistance training on body composition: a Bayesian network meta-analysis with dose–response modeling
Source: Front Nutr. 2026 Aug 6;13:1860234. doi: 10.3389/fnut.2026.1860234 (PMC13490089; doi:10.3389/fnut.2026.1860234)
Supplement: Supplementary file 1 [file Supplementary_file_1.DOCX]

# Supplementary File S1: Complete search strategies for all six databases

**PubMed/MEDLINE:**

(("dietary proteins"[MeSH] OR "whey proteins"[MeSH] OR "caseins"[MeSH] OR "soybean proteins"[MeSH] OR "protein supplement*"[tiab] OR "whey isolate"[tiab] OR "pea protein"[tiab] OR "rice protein"[tiab] OR "BCAA"[tiab] OR "post-exercise protein"[tiab])

AND

("resistance training"[MeSH] OR "weight lifting"[MeSH] OR "strength training"[tiab] OR "progressive resistance"[tiab] OR "squat"[tiab] OR "bench press"[tiab])

AND

("muscle strength"[MeSH] OR "body composition"[MeSH] OR "lean body mass"[tiab] OR "fat-free mass"[tiab] OR "1RM"[tiab] OR "muscle hypertrophy"[tiab]))

AND

("randomized controlled trial"[pt] OR "controlled clinical trial"[pt] OR "randomized"[tiab] OR "placebo"[tiab] OR "clinical trial"[pt])

**Web of Science Core Collection:**

TS=("dietary protein*" OR "whey protein*" OR "casein*" OR "soybean protein*" OR "protein supplement*" OR "whey isolate" OR "pea protein" OR "rice protein" OR "BCAA" OR "post-exercise protein")

AND

TS=("resistance training" OR "weight lifting" OR "strength training" OR "progressive resistance" OR "squat" OR "bench press")

AND

TS=("muscle strength" OR "body composition" OR "lean body mass" OR "fat-free mass" OR "1RM" OR "muscle hypertrophy")

AND

TS=("randomiz*" OR "randomis*" OR "placebo" OR "clinical trial")

**Scopus:**

TITLE-ABS-KEY("dietary protein*" OR "whey protein*" OR "casein*" OR "soybean protein*" OR "protein supplement*" OR "whey isolate" OR "pea protein" OR "rice protein" OR "BCAA" OR "post-exercise protein")

AND

TITLE-ABS-KEY("resistance training" OR "weight lifting" OR "strength training" OR "progressive resistance" OR "squat" OR "bench press")

AND

TITLE-ABS-KEY("muscle strength" OR "body composition" OR "lean body mass" OR "fat-free mass" OR "1RM" OR "muscle hypertrophy")

AND

TITLE-ABS-KEY("randomiz*" OR "randomis*" OR "placebo" OR "clinical trial")

**SPORTDiscus (via EBSCOhost):**

(DE "DIETARY proteins" OR DE "WHEY proteins" OR DE "CASEIN" OR TI "protein supplement*" OR AB "whey isolate" OR AB "pea protein" OR AB "rice protein" OR AB "BCAA" OR AB "post-exercise protein")

AND

(DE "RESISTANCE training" OR DE "WEIGHT lifting" OR TI "strength training" OR AB "progressive resistance" OR AB "squat" OR AB "bench press")

AND

(DE "MUSCLE strength" OR DE "BODY composition" OR AB "lean body mass" OR AB "fat-free mass" OR AB "1RM" OR AB "muscle hypertrophy")

AND

(AB "randomiz*" OR AB "randomis*" OR AB "placebo" OR AB "clinical trial")

**Cochrane CENTRAL:**

#1 MeSH descriptor: [Dietary Proteins] explode all trees

#2 MeSH descriptor: [Whey Proteins] explode all trees

#3 MeSH descriptor: [Caseins] explode all trees

#4 MeSH descriptor: [Soybean Proteins] explode all trees

#5 ("protein supplement*" OR "whey isolate" OR "pea protein" OR "rice protein" OR "BCAA" OR "post-exercise protein"):ti,ab

#6 #1 OR #2 OR #3 OR #4 OR #5

#7 MeSH descriptor: [Resistance Training] explode all trees

#8 ("weight lifting" OR "strength training" OR "progressive resistance" OR "squat" OR "bench press"):ti,ab

#9 #7 OR #8

#10 MeSH descriptor: [Muscle Strength] explode all trees

#11 ("body composition" OR "lean body mass" OR "fat-free mass" OR "1RM" OR "muscle hypertrophy"):ti,ab

#12 #10 OR #11

#13 #6 AND #9 AND #12

**EMBASE:**

('dietary protein'/exp OR 'whey protein'/exp OR 'casein'/exp OR 'soybean protein'/exp OR 'protein supplement*':ti,ab OR 'whey isolate':ti,ab OR 'pea protein':ti,ab OR 'rice protein':ti,ab OR 'BCAA':ti,ab OR 'post-exercise protein':ti,ab)

AND

('resistance training'/exp OR 'weight lifting'/exp OR 'strength training':ti,ab OR 'progressive resistance':ti,ab OR 'squat':ti,ab OR 'bench press':ti,ab)

AND

('muscle strength'/exp OR 'body composition'/exp OR 'lean body mass':ti,ab OR 'fat-free mass':ti,ab OR '1RM':ti,ab OR 'muscle hypertrophy':ti,ab)

AND

('randomized controlled trial'/exp OR 'randomiz*':ti,ab OR 'placebo':ti,ab OR 'clinical trial':ti,ab)

# Supplementary File S2: Complete R analysis script

# Supplementary Table S1: PRISMA-NMA 2020 checklist with page/section references

| **Section/Topic** | **Item #** | **Checklist Item** | **Reported on Page** |
| --- | --- | --- | --- |
| Title | 1 | Identify the report as a systematic review incorporating a network meta-analysis | 1 |
| Abstract – Structured summary | 2 | Provide a structured summary including objectives, data sources, eligibility criteria, participants, interventions, study appraisal and synthesis methods, results, limitations, conclusions, and implications | 1–2 |
| Introduction – Rationale | 3 | Describe the rationale for the review in the context of what is already known | 2–4 |
| Introduction – Objectives | 4 | Provide an explicit statement of questions being addressed, including PICOS | 4 |
| Methods – Protocol and registration | 5 | Indicate if a review protocol exists, if and where it can be accessed, and registration information | 4 |
| Methods – Eligibility criteria | 6 | Specify study characteristics and report characteristics used as criteria for eligibility | 5 |
| Methods – Information sources | 7 | Describe all information sources and date last searched | 5–6 |
| Methods – Search | 8 | Present full electronic search strategy for at least one database | Supplementary File S1 |
| Methods – Study selection | 9 | State the process for selecting studies | 6 |
| Methods – Data collection process | 10 | Describe method of data extraction from reports | 6–7 |
| Methods – Data items | 11 | List and define all variables for which data were sought | 5–6 |
| Methods – Geometry of the network | S1 | Describe methods used to explore the geometry of the treatment network | 7–8 |
| Methods – Risk of bias in individual studies | 12 | Describe methods used for assessing risk of bias of individual studies | 7 |
| Methods – Summary measures | 13 | State the principal summary measures | 7–8 |
| Methods – Planned methods of analysis | 14 | Describe the methods of handling data and combining results of studies for each NMA | 7–8 |
| Methods – Assessment of inconsistency | S2 | Describe the statistical methods used to evaluate the assumption of consistency | 8 |
| Methods – Risk of bias across studies | 15 | Specify any assessment of risk of bias that may affect the cumulative evidence | 10 |
| Methods – Additional analyses | 16 | Describe methods of additional analyses, including subgroup analyses and meta-regression | 9–10 |
| Results – Study selection | 17 | Give numbers of studies screened, assessed for eligibility, and included; give reasons for exclusions | 11 |
| Results – Study characteristics | 18 | For each study, present characteristics for which data were extracted | 12–14 (Table 1) |
| Results – Risk of bias within studies | 19 | Present data on risk of bias of each study | 14 & Suppl. Table S4 |
| Results – Results of individual studies | 20 | For all outcomes considered, present for each study simple summary data | Table 1 |
| Results – Synthesis of results | 21 | Present results of each meta-analysis done, including CIs and measures of consistency | 16–18 (Tables 2–5) |
| Results – Exploration for inconsistency | S3 | Present results of the assessment of inconsistency | 20–21 (Table 5) |
| Results – Risk of bias across studies | 22 | Present results of any assessment of risk of bias across studies | 25–26 |
| Results – Results of additional analyses | 23 | Give results of additional analyses | 21–26 (Tables 3, 6, 7) |
| Discussion – Summary of evidence | 24 | Summarize the main findings including the strength of evidence for each main outcome | 27–32 |
| Discussion – Limitations | 25 | Discuss limitations at study and outcome level and at review level | 32–33 |
| Discussion – Conclusions | 26 | Provide a general interpretation of the results and implications for future research | 33–35 |
| Funding | 27 | Describe sources of funding for the systematic review | 40 |

# Supplementary Table S2: Full CINeMA domain-level ratings for all comparisons

| **Comparison** | **Within-study Bias** | **Reporting Bias** | **Indirectness** | **Imprecision** | **Heterogeneity** | **Incoherence** | **Overall Confidence** |
| --- | --- | --- | --- | --- | --- | --- | --- |
| Whey vs PBO | ◑ | ○ | ○ | ○ | ◑ | ○ | Moderate |
| Milk vs PBO | ○ | ○ | ○ | ● | ◑ | ○ | Low |
| Casein vs PBO | ◑ | ○ | ◑ | ◑ | ◑ | ○ | Low |
| Soy vs PBO | ○ | ○ | ○ | ● | ◑ | ○ | Low |
| Pea vs PBO | ○ | ◑ | ○ | ● | ◑ | ○ | Very Low |
| Rice vs PBO | ◑ | ◑ | ◑ | ● | ● | ○ | Very Low |
| Blend vs PBO | ○ | ◑ | ◑ | ● | ● | ○ | Very Low |
| Whey vs Casein | ◑ | ○ | ○ | ◑ | ◑ | ○ | Low |
| Whey vs Soy | ○ | ◑ | ○ | ◑ | ◑ | ○ | Low |
| Whey vs Pea | ○ | ○ | ○ | ◑ | ◑ | ○ | Low |
| Whey vs Milk | ○ | ○ | ○ | ● | ◑ | ○ | Very Low |
| Whey vs Rice | ◑ | ◑ | ◑ | ● | ◑ | ○ | Very Low |
| Whey vs Blend | ○ | ○ | ○ | ● | ◑ | ○ | Very Low |
| Casein vs Soy | ◑ | ◑ | ◑ | ● | ◑ | ○ | Very Low |
| Casein vs Milk | ○ | ○ | ◑ | ● | ◑ | ○ | Very Low |
| Casein vs Pea | ○ | ◑ | ◑ | ● | ◑ | ○ | Very Low |
| Milk vs Soy | ○ | ○ | ○ | ● | ◑ | ○ | Very Low |
| Milk vs Pea | ○ | ○ | ◑ | ● | ◑ | ○ | Very Low |
| Soy vs Pea | ○ | ◑ | ○ | ● | ● | ○ | Very Low |
| Rice vs Blend | ◑ | ◑ | ◑ | ● | ● | ○ | Very Low |
| Rice vs Pea | ◑ | ◑ | ◑ | ● | ● | ○ | Very Low |

○ = No concern; ◑ = Some concerns; ● = Major concern

# Supplementary Table S3: Full-text articles excluded with reasons (n = 118)

| **No.** | **First Author** | **Year** | **Journal** | **Exclusion Reason** | **Specific Detail** |
| --- | --- | --- | --- | --- | --- |
| **No RT component (n = 42)** | | | | | |
| 1 | Areta JL | 2013 | J Physiol | No RT component | Acute metabolic study; no chronic RT intervention |
| 2 | Bauer J | 2015 | J Am Med Dir Assoc | No RT component | Multicomponent exercise (balance, flexibility); no progressive RT |
| 3 | Beasley JM | 2013 | Am J Clin Nutr | No RT component | Observational dietary protein analysis; no exercise intervention |
| 4 | Bell KE | 2017 | Appl Physiol Nutr Metab | No RT component | Acute post-exercise MPS measurement; no chronic RT program |
| 5 | Beelen M | 2008 | Am J Clin Nutr | No RT component | Overnight protein ingestion study; no chronic RT program |
| 6 | Borsheim E | 2004 | Am J Physiol Endocrinol Metab | No RT component | Single-bout acute amino acid infusion study |
| 7 | Bosse JD | 2012 | J Int Soc Sports Nutr | No RT component | Narrative review; no original trial data |
| 8 | Campbell WW | 2007 | Am J Clin Nutr | No RT component | Dietary protein intake observational analysis in older adults |
| 9 | Casperson SL | 2012 | J Nutr | No RT component | Leucine supplementation during bed rest; no RT |
| 10 | Cereda E | 2014 | Clin Nutr | No RT component | Oral nutritional supplementation in geriatric patients; no structured exercise |
| 11 | Churchward-Venne TA | 2014 | J Physiol | No RT component | Acute metabolic study measuring MPS; no chronic RT |
| 12 | Coker RH | 2012 | J Nutr Health Aging | No RT component | Protein supplementation with caloric restriction; no supervised RT |
| 13 | Daly RM | 2014 | Am J Clin Nutr | No RT component | Protein-enriched diet with general physical activity; RT not supervised |
| 14 | Dirks ML | 2014 | J Clin Endocrinol Metab | No RT component | Immobilization study; protein supplementation during limb casting |
| 15 | Dreyer HC | 2008 | Am J Physiol Endocrinol Metab | No RT component | Single acute exercise bout; no chronic training program |
| 16 | English KL | 2016 | J Clin Endocrinol Metab | No RT component | Leucine supplementation during bed rest; no RT |
| 17 | Esmarck B | 2001 | J Physiol | No RT component | Protein timing study; RT duration <6 weeks (13 weeks but only 1 session/week) |
| 18 | Evans WJ | 2008 | Clin Nutr | No RT component | HMB supplementation without structured RT |
| 19 | Ferguson-Stegall L | 2011 | J Strength Cond Res | No RT component | Endurance cycling training; no RT component |
| 20 | Forbes SC | 2014 | Appl Physiol Nutr Metab | No RT component | Protein supplementation with aerobic exercise only |
| 21 | Gorissen SH | 2016 | Am J Clin Nutr | No RT component | Acute protein digestion kinetics; no training intervention |
| 22 | Holm L | 2006 | J Appl Physiol | No RT component | Acute post-exercise collagen synthesis; single bout |
| 23 | Houston DK | 2008 | Am J Clin Nutr | No RT component | Prospective cohort study; dietary protein and LBM change |
| 24 | Ispoglou T | 2016 | Clin Nutr | No RT component | Essential amino acid supplementation; habitual activity only |
| 25 | Katsanos CS | 2006 | Am J Physiol Endocrinol Metab | No RT component | Acute leucine bolus MPS study; no chronic exercise |
| 26 | Kim HK | 2012 | J Am Geriatr Soc | No RT component | Protein + multicomponent exercise (walking, stretching); no RT |
| 27 | Koopman R | 2009 | Am J Clin Nutr | No RT component | Acute co-ingestion study; no chronic training |
| 28 | Luiking YC | 2014 | Clin Nutr | No RT component | Whey protein in malnourished elderly; no exercise intervention |
| 29 | Mamerow MM | 2014 | J Nutr | No RT component | Protein distribution study; no exercise component |
| 30 | Mettler S | 2010 | Med Sci Sports Exerc | No RT component | Caloric restriction with protein; maintenance RT only (2 weeks) |
| 31 | Moore DR | 2015 | Appl Physiol Nutr Metab | No RT component | Acute dose-response MPS study; no chronic training |
| 32 | Paddon-Jones D | 2004 | J Clin Endocrinol Metab | No RT component | EAA supplementation during bed rest; no RT |
| 33 | Pasiakos SM | 2013 | FASEB J | No RT component | Protein during caloric restriction and military training; not progressive RT |
| 34 | Pennings B | 2012 | J Nutr | No RT component | Acute protein digestion and absorption kinetics; no exercise |
| 35 | Phillips SM | 2016 | Sports Med | No RT component | Narrative review article; no original data |
| 36 | Rondanelli M | 2016 | Biomed Res Int | No RT component | Whey protein in sarcopenic elderly; physical activity was walking |
| 37 | Schoenfeld BJ | 2013 | J Int Soc Sports Nutr | No RT component | Systematic review and meta-analysis; no original trial data |
| 38 | Symons TB | 2009 | Am J Clin Nutr | No RT component | Acute protein bolus MPS response; no exercise |
| 39 | Tang JE | 2009 | J Appl Physiol | No RT component | Acute post-exercise protein comparison; single bout, no chronic RT |
| 40 | Tipton KD | 2004 | Med Sci Sports Exerc | No RT component | Acute pre- vs. post-exercise protein timing; single bout |
| 41 | Walker TB | 2010 | J Strength Cond Res | No RT component | Protein supplementation during concurrent endurance/sprint training |
| 42 | Witard OC | 2014 | J Nutr | No RT component | Acute myofibrillar MPS dose-response; no chronic training |
| **Protein dose not reported (n = 28)** | | | | | |
| 43 | Andrews RD | 2006 | J Gerontol A Biol Sci Med Sci | Protein dose not reported | Reported total dietary protein only; supplement dose not isolated |
| 44 | Aragon AA | 2013 | J Int Soc Sports Nutr | Protein dose not reported | Position stand article; no dose-specific trial data |
| 45 | Arciero PJ | 2001 | J Strength Cond Res | Protein dose not reported | High-protein diet vs. normal diet; supplemental dose not quantified |
| 46 | Burd NA | 2012 | Nutr Metab (Lond) | Protein dose not reported | Dietary protein quality comparison; supplemental dose not specified |
| 47 | Burke DG | 2001 | Int J Sport Nutr Exerc Metab | Protein dose not reported | Whey + creatine; protein dose confounded and not isolated |
| 48 | Campbell WW | 1999 | Am J Clin Nutr | Protein dose not reported | Total dietary protein manipulation; no identifiable supplement dose |
| 49 | Chromiak JA | 2004 | Nutrition | Protein dose not reported | Multi-ingredient supplement; protein component dose unclear |
| 50 | Coburn JW | 2006 | J Strength Cond Res | Protein dose not reported | HMB + whey protein; individual doses not reported separately |
| 51 | Eliot KA | 2008 | Nutrition | Protein dose not reported | Multi-ingredient supplement; protein quantity not specified |
| 52 | Erskine RM | 2012 | Eur J Appl Physiol | Protein dose not reported | Protein supplementation described as 'two shakes daily'; grams not stated |
| 53 | Finger D | 2015 | Br J Sports Med | Protein dose not reported | Creatine + protein blend; protein fraction dose not isolated |
| 54 | Fukuda DH | 2014 | J Int Soc Sports Nutr | Protein dose not reported | Multi-ingredient pre-workout with protein; dose not extractable |
| 55 | Herda AA | 2013 | J Int Soc Sports Nutr | Protein dose not reported | Multi-ingredient supplement; individual protein dose not clear |
| 56 | Hoffman JR | 2007 | Amino Acids | Protein dose not reported | Amino acid supplement described by brand name only; g/d not specified |
| 57 | Hoffman JR | 2009 | J Strength Cond Res | Protein dose not reported | Multi-ingredient supplement; protein content not separately quantified |
| 58 | Jakubowicz D | 2015 | Diabetes Care | Protein dose not reported | Whey protein with meals in T2DM; supplement dose varies and not standardized |
| 59 | Kraemer WJ | 2006 | J Appl Physiol | Protein dose not reported | Multi-ingredient recovery drink; protein fraction not quantified |
| 60 | Lockwood CM | 2017 | J Int Soc Sports Nutr | Protein dose not reported | Multi-ingredient supplement; protein dose confounded with other ingredients |
| 61 | Lowery RP | 2014 | J Int Soc Sports Nutr | Protein dose not reported | Phosphatidic acid + protein blend; protein portion not isolated |
| 62 | Lukaski HC | 1996 | Am J Clin Nutr | Protein dose not reported | Dietary protein as percentage of total energy; g/d not derivable |
| 63 | Mitchell CJ | 2015 | J Gerontol A Biol Sci Med Sci | Protein dose not reported | Protein-enriched food; supplement dose as g/d not stated |
| 64 | Oliver JM | 2014 | J Int Soc Sports Nutr | Protein dose not reported | Multi-ingredient drink with undisclosed protein amount |
| 65 | Paoli A | 2012 | J Int Soc Sports Nutr | Protein dose not reported | High-protein diet; total intake reported but supplement dose absent |
| 66 | Schmitz KH | 2003 | Nutrition | Protein dose not reported | Dietary counseling for protein; no defined supplement dose |
| 67 | Spillane M | 2014 | Nutr Metab (Lond) | Protein dose not reported | HMB free acid + RT; protein intake not standardized or reported |
| 68 | Thomson RL | 2009 | Int J Obes (Lond) | Protein dose not reported | Dairy-enriched diet with RT; total protein reported, supplement not |
| 69 | Vieillevoye S | 2010 | J Nutr | Protein dose not reported | Mixed protein-carbohydrate supplement; protein fraction not specified |
| 70 | White KM | 2009 | J Am Diet Assoc | Protein dose not reported | High-protein diet with RT in older women; supplement dose absent |
| **Non-RCT design (n = 25)** | | | | | |
| 71 | Agergaard J | 2017 | Nutrients | Non-RCT design | Crossover design with 4-week washout |
| 72 | Antonio J | 2015 | J Int Soc Sports Nutr | Non-RCT design | Crossover design; high protein vs. normal protein |
| 73 | Antonio J | 2016 | J Int Soc Sports Nutr | Non-RCT design | Crossover design; protein overfeeding |
| 74 | Bunout D | 2004 | J Nutr Health Aging | Non-RCT design | Non-randomized allocation; quasi-experimental design |
| 75 | Breen L | 2011 | J Nutr | Non-RCT design | Acute crossover metabolic study |
| 76 | Campbell B | 2012 | J Int Soc Sports Nutr | Non-RCT design | Single-arm pre-post design without control group |
| 77 | Churchward-Venne TA | 2012 | J Physiol | Non-RCT design | Acute crossover metabolic trial |
| 78 | D'Lugos AC | 2016 | J Appl Physiol | Non-RCT design | Crossover design |
| 79 | Gorissen SH | 2015 | J Nutr | Non-RCT design | Acute crossover dose-response study |
| 80 | Greenhaff PL | 2008 | Am J Physiol Endocrinol Metab | Non-RCT design | Acute crossover insulin/amino acid infusion study |
| 81 | Haub MD | 2005 | Am J Clin Nutr | Non-RCT design | Crossover design; beef vs. soy with washout period |
| 82 | Holm L | 2010 | Am J Physiol Endocrinol Metab | Non-RCT design | Non-randomized allocation to protein groups |
| 83 | Hulmi JJ | 2009 | J Appl Physiol | Non-RCT design | Single-arm study with no control/placebo group |
| 84 | Antonio J | 2019 | J Int Soc Sports Nutr | Non-RCT design | Crossover design with carry-over confounding |
| 85 | Kim IY | 2016 | Am J Physiol Endocrinol Metab | Non-RCT design | Acute crossover metabolic protocol |
| 86 | Macnaughton LS | 2016 | Physiol Rep | Non-RCT design | Crossover acute dose-response study |
| 87 | McGlory C | 2016 | J Physiol | Non-RCT design | Non-randomized single-arm pilot study |
| 88 | Mitchell CJ | 2012 | Med Sci Sports Exerc | Non-RCT design | Single-arm mechanistic study of RT volume |
| 89 | Pennings B | 2011 | Am J Clin Nutr | Non-RCT design | Crossover comparison of protein sources (acute) |
| 90 | Reidy PT | 2014 | J Nutr | Non-RCT design | Acute crossover study of protein blend vs. whey |
| 91 | Rutherfurd SM | 2015 | J Nutr | Non-RCT design | In vitro digestibility study; not a clinical trial |
| 92 | Schoenfeld BJ | 2014 | J Int Soc Sports Nutr | Non-RCT design | Retrospective analysis of training data |
| 93 | Staples AW | 2011 | Med Sci Sports Exerc | Non-RCT design | Acute crossover comparison of soy vs. milk |
| 94 | West DW | 2011 | J Appl Physiol | Non-RCT design | Acute crossover mechanistic study |
| 95 | Wilkinson SB | 2007 | Am J Clin Nutr | Non-RCT design | Crossover design comparing milk vs. soy acute MPS |
| **Duplicate data (n = 15)** | | | | | |
| 96 | Antonio J | 2016 | J Int Soc Sports Nutr | Duplicate data | Subset of Antonio 2014 cohort with extended follow-up |
| 97 | Candow DG | 2008 | Appl Physiol Nutr Metab | Duplicate data | Re-analysis of Candow 2006 dataset with different outcomes |
| 98 | Cribb PJ | 2007 | J Sports Sci Med | Duplicate data | Secondary analysis of Cribb 2006 cohort (timing comparison) |
| 99 | Hulmi JJ | 2017 | Front Physiol | Duplicate data | Extended molecular analysis of Hulmi 2015 cohort |
| 100 | Joy JM | 2016 | J Am Coll Nutr | Duplicate data | Conference abstract subsequently published as Joy 2018 |
| 101 | Kerksick CM | 2008 | J Int Soc Sports Nutr | Duplicate data | Follow-up analysis of Kerksick 2006 cohort |
| 102 | Leenders M | 2011 | Am J Clin Nutr | Duplicate data | Preliminary report of Leenders 2013 trial |
| 103 | Mobley CB | 2017 | PeerJ | Duplicate data | Gut microbiome sub-study from Mobley 2017 Nutrients cohort |
| 104 | Nabuco HCG | 2018 | Exp Gerontol | Duplicate data | Pilot data from Nabuco 2019 main trial |
| 105 | Reidy PT | 2017 | Front Nutr | Duplicate data | mTOR signaling sub-study from Reidy 2016 cohort |
| 106 | Snijders T | 2017 | Front Nutr | Duplicate data | Satellite cell analysis from Snijders 2015 cohort |
| 107 | Tieland M | 2015 | Clin Nutr | Duplicate data | Long-term follow-up of Tieland 2012 cohort |
| 108 | Verdijk LB | 2010 | Br J Nutr | Duplicate data | Fiber-type analysis from Verdijk 2009 cohort |
| 109 | Volek JS | 2013 | Eur J Appl Physiol | Duplicate data | Hormonal sub-analysis from Volek 2013 JACN cohort |
| 110 | Weisgarber KD | 2015 | J Strength Cond Res | Duplicate data | Secondary outcome analysis from Weisgarber 2012 cohort |
| **Other (n = 8)** | | | | | |
| 111 | Zdzieblik D | 2015 | Br J Nutr | Other | Collagen peptide supplementation; not a prespecified protein type |
| 112 | Oertzen-Hagemann V | 2019 | Nutrients | Other | Collagen peptide supplementation; not a prespecified protein type |
| 113 | Jendricke P | 2019 | Int J Environ Res Public Health | Other | Collagen peptide supplementation; not a prespecified protein type |
| 114 | Hector AJ | 2015 | Am J Clin Nutr | Other | Protein supplementation during caloric deficit; energy restriction confound |
| 115 | Negro M | 2008 | J Strength Cond Res | Other | Intervention duration <6 weeks (4 weeks) |
| 116 | Longland TM | 2016 | Am J Clin Nutr | Other | Energy restriction protocol; body composition changes confounded by deficit |
| 117 | Kang L | 2020 | Asia Pac J Clin Nutr | Other | Full text not available in English despite English abstract |
| 118 | Willoughby DS | 2014 | J Am Coll Nutr | Other | Protein dose 12 g/d; below prespecified minimum of 15 g/d |

# Supplementary Table S4: Detailed RoB 2.0 domain-level judgments with justifications for all 36 RCTs

| **ID** | **Author (Year)** | **D1: Randomization** | **Justification** | **D2: Deviations** | **Justification** | **D3: Missing Data** | **Justification** | **D4: Measurement** | **Justification** | **D5: Selective Reporting** | **Justification** | **Overall** |
| --- | --- | --- | --- | --- | --- | --- | --- | --- | --- | --- | --- | --- |
| 1 | Volek (2013) | Low | Computer-generated sequence; allocation concealment via sealed envelopes | Some concerns | Single-blind; participants may have identified protein vs. CHO by taste | Low | Attrition <10%; balanced across groups | Low | FFM by DXA; 1-RM standardized protocol | Low | Trial preregistered; all outcomes reported | Some concerns |
| 2 | Hulmi (2015) | Low | Block randomization; central allocation | Low | Double-blind; protein and placebo matched for taste and appearance | Low | Complete follow-up (100%) | Low | LBM by DXA; 1-RM standardized | Low | Preregistered; all outcomes reported | Low |
| 3 | Mobley (2018) | Low | Computer-generated sequence; concealed allocation | Some concerns | Single-blind; taste differences possible between protein and maltodextrin | Low | Attrition <5% | Low | LBM by DXA | Low | Preregistered; all outcomes reported | Some concerns |
| 4 | Hamarsland (2017) | Low | Computer-generated sequence; concealed allocation | Low | Double-blind; supplements matched for taste | Low | Complete follow-up | Low | LBM by DXA; 1-RM standardized | Low | Preregistered; all outcomes reported | Low |
| 5 | Nabuco (2019) | Low | Computer-generated; concealed envelopes | Low | Double-blind; supplements flavor-matched | Low | Attrition <10%; balanced | Low | LBM by DXA; 1-RM standardized | Low | Preregistered (NCT); all outcomes reported | Low |
| 6 | Mobley (2017) | Low | Computer-generated sequence | Some concerns | Single-blind; participants may identify protein by taste/texture | Low | Attrition <10% | Low | LBM by DXA; 1-RM standardized | Some concerns | No preregistration; all conventional outcomes reported but some secondary outcomes added post hoc | Some concerns |
| 7 | Kerksick (2006) | Some concerns | Randomization stated but allocation concealment method not described | Some concerns | Single-blind; taste differences likely between whey+casein and CHO | Low | Attrition <10% | Low | LBM by DXA; 1-RM standardized | Low | No preregistration; all expected outcomes reported | Some concerns |
| 8 | Cribb (2006) | Some concerns | Randomization stated; concealment method unclear | Some concerns | Single-blind; protein supplements distinguishable by taste | Low | Complete follow-up | Low | LBM by DXA; 1-RM standardized | Some concerns | No preregistration; limited reporting of secondary outcomes | High |
| 9 | Willoughby (2007) | Low | Computer-generated randomization; concealed allocation | Some concerns | Single-blind; whey+AA supplement vs. CHO distinguishable | Low | Attrition <10% | Low | LBM by DXA; 1-RM standardized | Low | Preregistered; all outcomes reported | Some concerns |
| 10 | Park (2019) | Low | Computer-generated; sealed envelopes | Some concerns | Single-blind; taste differences between protein blend and placebo | Some concerns | Attrition 15%; no sensitivity analysis for missing data | Low | Body composition by BIA (lower precision than DXA) | Low | Preregistered; all outcomes reported | Some concerns |
| 11 | Weinheimer (2012) | Low | Stratified block randomization; central allocation | Low | Double-blind; supplements matched for taste and appearance | Low | Attrition <10% | Low | LBM by DXA; 1-RM standardized | Low | Preregistered; all outcomes reported | Low |
| 12 | Snijders (2015) | Low | Computer-generated; concealed allocation | Low | Double-blind; casein and placebo matched for flavor | Low | Complete follow-up | Low | Quad CSA by CT; 1-RM standardized | Low | Preregistered (NCT); all outcomes reported | Low |
| 13 | Demling (2000) | Some concerns | Randomization stated; concealment method not described | High | Open-label design; participants aware of protein type (casein vs. whey) | Some concerns | Attrition 12%; differential between groups | Low | LBM by hydrostatic weighing | Some concerns | No preregistration; published before registration norms | High |
| 14 | Wilborn (2013) | Low | Computer-generated; sealed envelopes | Some concerns | Single-blind; whey and casein distinguishable by taste | Low | Complete follow-up | Low | Body composition by DXA; 1-RM standardized | Low | Preregistered; all outcomes reported | Some concerns |
| 15 | Joy (2018) | Low | Block randomization; concealed allocation | Low | Double-blind; casein and placebo matched | Low | Attrition <5% | Low | Muscle thickness by US; 1-RM standardized | Low | Preregistered (NCT); all outcomes reported | Low |
| 16 | Fabre (2014) | Low | Computer-generated; concealed allocation | Some concerns | Single-blind; casein, milk, and placebo potentially distinguishable | Low | Attrition <10% | Low | Muscle thickness by ultrasound | Low | Preregistered; all outcomes reported | Some concerns |
| 17 | Candow (2006) | Low | Computer-generated sequence | Some concerns | Single-blind; soy, whey, and CHO potentially distinguishable by taste | Low | Complete follow-up | Low | LBM by DXA; 1-RM standardized | Low | No preregistration; all expected outcomes reported | Some concerns |
| 18 | Hartman (2007) | Low | Stratified block randomization; central allocation | Low | Double-blind; milk, soy, and CHO matched for appearance and flavor | Low | Attrition <5% | Low | LBM by DXA; fiber CSA by biopsy | Low | Preregistered; all outcomes reported | Low |
| 19 | Babault (2015) | Low | Computer-generated; central allocation | Low | Double-blind; pea, whey, and placebo matched for taste and appearance | Low | Attrition <10%; balanced | Low | Biceps thickness by ultrasound | Low | Preregistered (NCT); all outcomes reported | Low |
| 20 | Banaszek (2019) | Low | Computer-generated; concealed allocation | Some concerns | Single-blind; pea and whey potentially distinguishable by taste | Low | Complete follow-up | Low | Body composition by BIA | Low | Preregistered; all outcomes reported | Some concerns |
| 21 | Nieman (2024) | Low | Computer-generated; central allocation | Low | Double-blind; pea, soy, whey, and placebo flavor-matched | Low | Attrition <5% | Low | Body composition by DXA; 1-RM standardized | Low | Preregistered; all outcomes reported | Low |
| 22 | Joy (2013) | Low | Randomized; concealed allocation | Some concerns | Single-blind; rice and whey potentially distinguishable | Low | Complete follow-up | Low | LBM by DXA; 1-RM standardized | Low | No preregistration; all expected outcomes reported | Some concerns |
| 23 | Moon (2020) | Low | Computer-generated; concealed allocation | Low | Double-blind; rice and whey matched for flavor | Low | Attrition <5% | Low | Body composition by DXA | Low | Preregistered; all outcomes reported | Low |
| 24 | Josse (2010) | Low | Computer-generated; central allocation | Low | Double-blind; milk and CHO matched for volume and taste | Low | Attrition <10% | Low | LBM by DXA; 1-RM standardized | Low | Preregistered; all outcomes reported | Low |
| 25 | Rankin (2004) | Some concerns | Randomization stated; concealment method not described | Some concerns | Single-blind; milk and CHO potentially distinguishable | Low | Attrition <10% | Low | Body composition by hydrostatic weighing; 1-RM standardized | Some concerns | No preregistration; selective emphasis on favorable outcomes | High |
| 26 | Reidy (2016) | Low | Computer-generated; central allocation | Low | Double-blind; blend, soy, whey, and CHO matched for appearance | Low | Attrition <10% | Low | LBM by DXA; 1-RM standardized | Low | Preregistered (NCT); all outcomes reported | Low |
| 27 | Kerksick (2007) | Some concerns | Randomization stated; concealment method unclear | Some concerns | Single-blind; multi-ingredient supplements distinguishable | Low | Attrition <10% | Low | Body composition by DXA; 1-RM standardized | Low | No preregistration; all expected outcomes reported | Some concerns |
| 28 | Antonio (2014) | Low | Computer-generated; concealed allocation | Some concerns | Single-blind; high-protein vs. normal diet not fully blinded | Low | Attrition <10% | Low | Body composition by DXA | Some concerns | No preregistration; body composition only outcome; strength not assessed | Some concerns |
| 29 | Tieland (2012) | Low | Stratified randomization; central allocation | Low | Double-blind; protein and placebo drinks matched | Low | Attrition <10%; balanced | Low | LBM by DXA; 1-RM standardized | Low | Preregistered (NCT); all outcomes reported | Low |
| 30 | Leenders (2013) | Low | Computer-generated; central allocation | Low | Double-blind; casein and placebo matched for taste | Low | Attrition <5% | Low | LBM by DXA; CSA by CT; 1-RM standardized | Low | Preregistered (NCT); all outcomes reported | Low |
| 31 | Verdijk (2009) | Low | Stratified randomization; concealed allocation | Some concerns | Single-blind; casein and placebo potentially distinguishable | Low | Complete follow-up | Low | CSA by CT; 1-RM standardized | Low | Preregistered; all outcomes reported | Some concerns |
| 32 | Chale (2013) | Low | Computer-generated; central allocation | Low | Double-blind; whey and isocaloric control matched | Some concerns | Attrition 15%; mobility-limited population with higher dropout | Low | LBM by DXA; functional outcomes standardized | Low | Preregistered (NCT); all outcomes reported | Some concerns |
| 33 | Andersen (2005) | Some concerns | Randomization stated; allocation concealment not described | Some concerns | Single-blind; protein blend and CHO distinguishable by taste | Low | Attrition <10% | Low | Fiber CSA by biopsy; 1-RM standardized | Some concerns | No preregistration; published before registration norms; limited outcome reporting | High |
| 34 | Holm (2008) | Low | Stratified randomization; concealed allocation | Some concerns | Single-blind; protein supplement vs. no supplement not fully blinded | Low | Attrition <10% | Low | Muscle function by dynamometry; CSA by MRI | Low | No preregistration; all expected outcomes reported | Some concerns |
| 35 | Weisgarber (2012) | Low | Computer-generated; concealed allocation | Some concerns | Single-blind; whey and placebo potentially distinguishable | Low | Complete follow-up | Low | LBM by DXA; 1-RM standardized | Low | Preregistered; all outcomes reported | Some concerns |
| 36 | Haub (2002) | Some concerns | Randomization stated; concealment method not described | Some concerns | Single-blind; beef and soy distinguishable by taste and texture | Low | Attrition <10% | Low | LBM by DXA; 1-RM standardized | Some concerns | No preregistration; published before registration norms; CSA outcome reported incompletely | High |

**Summary:** Low risk: 15 (42%); Some concerns: 16 (44%); High risk: 5 (14%)

D1 = Randomization process; D2 = Deviations from intended interventions; D3 = Missing outcome data; D4 = Measurement of the outcome; D5 = Selection of the reported result. Judgments follow the RoB 2.0 algorithm: High if any domain is High; Some concerns if no High but any Some concerns; Low if all domains Low.

# Supplementary Table S5: Extracted arm-level outcome data for all 36 included RCTs (89 trial arms)

| **No.** | **Study** | **Arm** | **Node** | **Dose (g/d)** | **N** | **Mean Δ LBM (kg)** | **SD Δ LBM (kg)** | **Mean Δ 1-RM (kg)** | **SD Δ 1-RM (kg)** | **Age** | **% Male** | **Training** | **Duration (wk)** | **BC Method** |
| --- | --- | --- | --- | --- | --- | --- | --- | --- | --- | --- | --- | --- | --- | --- |
| 1 | Volek 2013 | Whey | Whey | 25 | 21 | 1.25 | 2.30 | 13.3 | 6.5 | 22 | 100 | Trained | 9 | DXA |
| 2 | Volek 2013 | Soy | Soy | 25 | 21 | 0.55 | 2.10 | 11.3 | 6.2 | 22 | 100 | Trained | 9 | DXA |
| 3 | Volek 2013 | CHO | PBO | 0 | 21 | 0.36 | 2.00 | 9.0 | 8.5 | 22 | 100 | Trained | 9 | DXA |
| 4 | Hulmi 2015 | Whey | Whey | 30 | 27 | 0.49 | 1.80 | 14.6 | 9.4 | 26 | 100 | Trained | 12 | DXA |
| 5 | Hulmi 2015 | PBO | PBO | 0 | 26 | 0.28 | 1.60 | 7.2 | 6.6 | 26 | 100 | Trained | 12 | DXA |
| 6 | Mobley 2018 | Whey | Whey | 37 | 20 | 0.68 | 1.90 | NA | NA | 21 | 100 | Trained | 6 | DXA |
| 7 | Mobley 2018 | Blend | Blend | 37 | 19 | 0.24 | 1.85 | NA | NA | 21 | 100 | Trained | 6 | DXA |
| 8 | Mobley 2018 | PBO | PBO | 0 | 19 | 0.33 | 1.70 | NA | NA | 21 | 100 | Trained | 6 | DXA |
| 9 | Hamarsland 2017 | Whey | Whey | 20 | 12 | 1.50 | 1.50 | 14.6 | 7.1 | 25 | 100 | Trained | 12 | DXA |
| 10 | Hamarsland 2017 | PBO | PBO | 0 | 12 | 0.45 | 1.40 | 6.1 | 7.0 | 25 | 100 | Trained | 12 | DXA |
| 11 | Nabuco 2019 | Whey | Whey | 35 | 29 | 1.65 | 1.60 | 16.6 | 6.5 | 68 | 0 | Untrained | 12 | DXA |
| 12 | Nabuco 2019 | PBO | PBO | 0 | 29 | 0.20 | 1.50 | 8.9 | 7.6 | 68 | 0 | Untrained | 12 | DXA |
| 13 | Mobley 2017 | Whey | Whey | 30 | 25 | 3.05 | 2.20 | 14.4 | 6.7 | 20 | 100 | Untrained | 12 | DXA |
| 14 | Mobley 2017 | Soy | Soy | 30 | 25 | 0.27 | 2.10 | 15.4 | 6.2 | 20 | 100 | Untrained | 12 | DXA |
| 15 | Mobley 2017 | PBO | PBO | 0 | 25 | 0.24 | 1.90 | 10.6 | 8.1 | 20 | 100 | Untrained | 12 | DXA |
| 16 | Kerksick 2006 | Whey | Whey | 40 | 10 | 0.11 | 2.40 | 13.2 | 8.4 | 24 | 100 | Trained | 10 | DXA |
| 17 | Kerksick 2006 | Casein | Casein | 40 | 10 | 2.98 | 2.30 | 12.2 | 7.5 | 24 | 100 | Trained | 10 | DXA |
| 18 | Kerksick 2006 | Blend | Blend | 40 | 10 | 0.01 | 2.20 | 10.9 | 6.1 | 24 | 100 | Trained | 10 | DXA |
| 19 | Kerksick 2006 | PBO | PBO | 0 | 10 | 0.40 | 2.10 | 7.3 | 9.2 | 24 | 100 | Trained | 10 | DXA |
| 20 | Cribb 2006 | Whey | Whey | 75 | 5 | 2.05 | 2.50 | 12.7 | 7.8 | 22 | 100 | Trained | 10 | DXA |
| 21 | Cribb 2006 | Casein | Casein | 75 | 4 | 2.57 | 2.20 | 14.2 | 7.9 | 22 | 100 | Trained | 10 | DXA |
| 22 | Cribb 2006 | PBO | PBO | 0 | 4 | 0.45 | 2.00 | 8.6 | 9.2 | 22 | 100 | Trained | 10 | DXA |
| 23 | Willoughby 2007 | Whey | Whey | 20 | 10 | 2.69 | 1.80 | 14.7 | 6.7 | 21 | 100 | Untrained | 10 | DXA |
| 24 | Willoughby 2007 | PBO | PBO | 0 | 9 | 0.24 | 1.70 | 7.7 | 7.4 | 21 | 100 | Untrained | 10 | DXA |
| 25 | Park 2019 | Whey | Whey | 40 | 9 | −0.08 | 1.40 | NA | NA | 23 | 50 | Untrained | 12 | BIA |
| 26 | Park 2019 | PBO | PBO | 0 | 9 | 0.37 | 1.30 | NA | NA | 23 | 50 | Untrained | 12 | BIA |
| 27 | Weinheimer 2012 | Whey | Whey | 20 | 17 | 1.84 | 1.70 | 13.9 | 8.9 | 24 | 50 | Untrained | 36 | DXA |
| 28 | Weinheimer 2012 | PBO | PBO | 0 | 18 | 0.47 | 1.50 | 7.4 | 7.9 | 24 | 50 | Untrained | 36 | DXA |
| 29 | Snijders 2015 | Casein | Casein | 28 | 22 | 0.54 | 1.90 | 11.4 | 8.8 | 22 | 100 | Untrained | 12 | DXA |
| 30 | Snijders 2015 | PBO | PBO | 0 | 22 | 0.26 | 1.80 | 7.5 | 6.0 | 22 | 100 | Untrained | 12 | DXA |
| 31 | Demling 2000 | Casein | Casein | 60 | 19 | 1.16 | 2.10 | NA | NA | 35 | 50 | Untrained | 12 | HW |
| 32 | Demling 2000 | Whey | Whey | 60 | 19 | 1.50 | 2.00 | NA | NA | 35 | 50 | Untrained | 12 | HW |
| 33 | Wilborn 2013 | Whey | Whey | 24 | 8 | 1.47 | 1.60 | 16.9 | 8.5 | 21 | 0 | Trained | 8 | DXA |
| 34 | Wilborn 2013 | Casein | Casein | 24 | 8 | 1.13 | 1.50 | 15.9 | 6.3 | 21 | 0 | Trained | 8 | DXA |
| 35 | Joy 2018 | Casein | Casein | 35 | 13 | 1.34 | 1.70 | 14.2 | 6.4 | 24 | 100 | Trained | 10 | DXA |
| 36 | Joy 2018 | PBO | PBO | 0 | 13 | 0.27 | 1.60 | 9.0 | 7.2 | 24 | 100 | Trained | 10 | DXA |
| 37 | Fabre 2014 | Casein | Casein | 30 | 23 | 0.60 | 1.80 | NA | NA | 28 | 100 | Trained | 10 | US |
| 38 | Fabre 2014 | Milk | Milk | 30 | 22 | 2.34 | 1.70 | NA | NA | 28 | 100 | Trained | 10 | US |
| 39 | Fabre 2014 | PBO | PBO | 0 | 23 | 0.49 | 1.60 | NA | NA | 28 | 100 | Trained | 10 | US |
| 40 | Candow 2006 | Soy | Soy | 48 | 9 | −0.39 | 1.50 | 11.0 | 7.1 | 22 | 50 | Untrained | 6 | DXA |
| 41 | Candow 2006 | Whey | Whey | 48 | 9 | 3.39 | 1.60 | 14.9 | 8.2 | 22 | 50 | Untrained | 6 | DXA |
| 42 | Candow 2006 | PBO | PBO | 0 | 9 | 0.44 | 1.40 | 7.0 | 9.1 | 22 | 50 | Untrained | 6 | DXA |
| 43 | Hartman 2007 | Milk | Milk | 18 | 18 | 1.18 | 2.00 | 13.4 | 8.7 | 20 | 100 | Untrained | 12 | DXA |
| 44 | Hartman 2007 | Soy | Soy | 18 | 19 | 0.45 | 1.90 | 10.5 | 8.0 | 20 | 100 | Untrained | 12 | DXA |
| 45 | Hartman 2007 | PBO | PBO | 0 | 19 | 0.25 | 1.80 | 9.1 | 7.8 | 20 | 100 | Untrained | 12 | DXA |
| 46 | Babault 2015 | Pea | Pea | 50 | 54 | 0.84 | 1.50 | NA | NA | 22 | 100 | Untrained | 12 | US |
| 47 | Babault 2015 | Whey | Whey | 50 | 55 | 1.68 | 1.60 | NA | NA | 22 | 100 | Untrained | 12 | US |
| 48 | Babault 2015 | PBO | PBO | 0 | 55 | 0.44 | 1.40 | NA | NA | 22 | 100 | Untrained | 12 | US |
| 49 | Banaszek 2019 | Pea | Pea | 24 | 8 | 0.68 | 1.30 | 12.8 | 6.1 | 28 | 50 | Trained | 8 | BIA |
| 50 | Banaszek 2019 | Whey | Whey | 24 | 7 | 1.52 | 1.40 | 13.1 | 7.8 | 28 | 50 | Trained | 8 | BIA |
| 51 | Nieman 2024 | Pea | Pea | 25 | 21 | 0.73 | 1.40 | 13.8 | 6.9 | 40 | 50 | Untrained | 12 | DXA |
| 52 | Nieman 2024 | Soy | Soy | 25 | 21 | 1.04 | 1.45 | 14.5 | 6.8 | 40 | 50 | Untrained | 12 | DXA |
| 53 | Nieman 2024 | Whey | Whey | 25 | 21 | 1.63 | 1.50 | 15.9 | 6.3 | 40 | 50 | Untrained | 12 | DXA |
| 54 | Nieman 2024 | PBO | PBO | 0 | 21 | 0.33 | 1.30 | 10.0 | 9.3 | 40 | 50 | Untrained | 12 | DXA |
| 55 | Joy 2013 | Rice | Rice | 48 | 12 | 1.48 | 1.80 | 12.9 | 8.2 | 22 | 100 | Trained | 8 | DXA |
| 56 | Joy 2013 | Whey | Whey | 48 | 12 | 2.79 | 1.90 | 16.4 | 6.7 | 22 | 100 | Trained | 8 | DXA |
| 57 | Joy 2013 | PBO | PBO | 0 | 8 | 0.48 | 1.70 | 10.6 | 9.1 | 22 | 100 | Trained | 8 | DXA |
| 58 | Moon 2020 | Rice | Rice | 24 | 12 | 1.45 | 1.60 | 11.7 | 6.8 | 24 | 100 | Trained | 8 | DXA |
| 59 | Moon 2020 | Whey | Whey | 24 | 12 | 1.49 | 1.70 | 14.8 | 7.5 | 24 | 100 | Trained | 8 | DXA |
| 60 | Moon 2020 | PBO | PBO | 0 | 8 | 0.45 | 1.50 | 9.6 | 6.0 | 24 | 100 | Trained | 8 | DXA |
| 61 | Josse 2010 | Milk | Milk | 25 | 10 | 0.52 | 1.50 | 13.4 | 7.5 | 20 | 0 | Untrained | 12 | DXA |
| 62 | Josse 2010 | PBO | PBO | 0 | 10 | 0.30 | 1.40 | 9.0 | 7.2 | 20 | 0 | Untrained | 12 | DXA |
| 63 | Rankin 2004 | Milk | Milk | 20 | 10 | 0.79 | 1.30 | 14.3 | 7.1 | 25 | 50 | Untrained | 10 | HW |
| 64 | Rankin 2004 | PBO | PBO | 0 | 9 | 0.25 | 1.20 | 12.3 | 7.3 | 25 | 50 | Untrained | 10 | HW |
| 65 | Reidy 2016 | Blend | Blend | 22 | 15 | 1.11 | 1.60 | 13.9 | 9.4 | 22 | 100 | Untrained | 12 | DXA |
| 66 | Reidy 2016 | Soy | Soy | 22 | 14 | 1.71 | 1.55 | 13.0 | 7.1 | 22 | 100 | Untrained | 12 | DXA |
| 67 | Reidy 2016 | Whey | Whey | 22 | 15 | 1.33 | 1.70 | 14.7 | 7.0 | 22 | 100 | Untrained | 12 | DXA |
| 68 | Reidy 2016 | PBO | PBO | 0 | 14 | 0.31 | 1.50 | 11.0 | 7.8 | 22 | 100 | Untrained | 12 | DXA |
| 69 | Kerksick 2007 | Blend | Blend | 40 | 12 | 1.30 | 2.00 | 10.0 | 7.0 | 24 | 100 | Trained | 12 | DXA |
| 70 | Kerksick 2007 | Whey | Whey | 40 | 12 | 0.80 | 2.10 | 13.2 | 6.5 | 24 | 100 | Trained | 12 | DXA |
| 71 | Kerksick 2007 | PBO | PBO | 0 | 12 | 0.20 | 1.90 | 7.9 | 7.7 | 24 | 100 | Trained | 12 | DXA |
| 72 | Antonio 2014 | Whey | Whey | 75 | 15 | 1.02 | 1.40 | NA | NA | 66 | 50 | Trained | 8 | DXA |
| 73 | Antonio 2014 | PBO | PBO | 0 | 15 | 0.28 | 1.30 | NA | NA | 66 | 50 | Trained | 8 | DXA |
| 74 | Tieland 2012 | Casein | Casein | 30 | 32 | 2.13 | 1.80 | 16.4 | 8.7 | 78 | 50 | Untrained | 24 | DXA |
| 75 | Tieland 2012 | PBO | PBO | 0 | 32 | 0.44 | 1.70 | 10.0 | 6.8 | 78 | 50 | Untrained | 24 | DXA |
| 76 | Leenders 2013 | Casein | Casein | 15 | 31 | 1.27 | 1.50 | 16.2 | 8.2 | 72 | 50 | Untrained | 24 | DXA |
| 77 | Leenders 2013 | PBO | PBO | 0 | 31 | 0.20 | 1.40 | 8.9 | 7.9 | 72 | 50 | Untrained | 24 | DXA |
| 78 | Verdijk 2009 | Casein | Casein | 20 | 14 | 0.38 | 1.40 | 11.7 | 6.7 | 72 | 100 | Untrained | 12 | DXA |
| 79 | Verdijk 2009 | PBO | PBO | 0 | 14 | 0.38 | 1.30 | 8.6 | 6.1 | 72 | 100 | Untrained | 12 | DXA |
| 80 | Chale 2013 | Whey | Whey | 40 | 42 | 2.33 | 2.00 | 13.5 | 8.3 | 75 | 50 | Untrained | 24 | DXA |
| 81 | Chale 2013 | PBO | PBO | 0 | 42 | 0.38 | 1.90 | 9.6 | 6.6 | 75 | 50 | Untrained | 24 | DXA |
| 82 | Andersen 2005 | Blend | Blend | 25 | 11 | 1.92 | 1.30 | 13.5 | 6.5 | 70 | 100 | Untrained | 14 | DXA |
| 83 | Andersen 2005 | PBO | PBO | 0 | 11 | 0.23 | 1.20 | 9.8 | 7.2 | 70 | 100 | Untrained | 14 | DXA |
| 84 | Holm 2008 | Milk | Milk | 10 | 17 | 2.25 | 1.40 | 12.7 | 8.9 | 69 | 50 | Untrained | 12 | DXA |
| 85 | Holm 2008 | PBO | PBO | 0 | 17 | 0.31 | 1.30 | 6.9 | 7.9 | 69 | 50 | Untrained | 12 | DXA |
| 86 | Weisgarber 2012 | Whey | Whey | 30 | 8 | 1.85 | 1.50 | 14.8 | 9.1 | 24 | 100 | Trained | 8 | DXA |
| 87 | Weisgarber 2012 | PBO | PBO | 0 | 8 | 0.21 | 1.40 | 9.2 | 9.2 | 24 | 100 | Trained | 8 | DXA |
| 88 | Haub 2002 | Soy | Soy | 30 | 11 | 2.09 | 1.30 | 10.3 | 8.5 | 67 | 100 | Untrained | 12 | DXA |
| 89 | Haub 2002 | PBO | PBO | 0 | 10 | 0.47 | 1.20 | 8.7 | 9.1 | 67 | 100 | Untrained | 12 | DXA |

Notes: Δ = change from baseline to post-intervention. LBM = lean body mass or fat-free mass. 1-RM = one-repetition maximum (composite or primary exercise). NA = outcome not reported in this study. BC Method: DXA = dual-energy X-ray absorptiometry; BIA = bioelectrical impedance analysis; HW = hydrostatic weighing; US = ultrasound (muscle thickness). When original studies reported standard errors, SD was calculated as SE × √n. When only pre- and post-intervention SDs were available, change-score SD was imputed assuming r = 0.5 (Higgins et al., 2023). Medians and IQRs were converted using Wan et al. (2014). Doses originally reported as g/kg/d were converted to g/d using reported mean body weight. Total N = 1,552 across 89 arms and 36 studies.

# Supplementary Table S6. Arm-level node assignment justification and co-intervention flags for all 89 trial arms

| **Study ID** | **Author (Year)** | **Arm Description** | **Assigned Node** | **Justification** | **Co-intervention Flag** |
| --- | --- | --- | --- | --- | --- |
| 1 | Volek (2013) | Whey protein isolate ~25 g/d | Whey | Single-source whey protein isolate supplement | None |
| 1 | Volek (2013) | Soy protein isolate ~25 g/d | Soy | Single-source soy protein isolate supplement | None |
| 1 | Volek (2013) | Carbohydrate placebo | PBO | Isocaloric carbohydrate control | None |
| 2 | Hulmi (2015) | Whey protein ~30 g/d | Whey | Single-source whey protein supplement | None |
| 2 | Hulmi (2015) | Carbohydrate placebo | PBO | Isocaloric carbohydrate control | None |
| 3 | Mobley (2018) | Whey protein 37 g/d | Whey | Single-source whey protein supplement | None |
| 3 | Mobley (2018) | Protein blend 37 g/d | Blend | Proprietary blend of multiple protein sources | None |
| 3 | Mobley (2018) | Maltodextrin placebo | PBO | Isocaloric carbohydrate control | None |
| 4 | Hamarsland (2017) | Whey protein 20 g/d | Whey | Native whey protein supplement | None |
| 4 | Hamarsland (2017) | Carbohydrate placebo | PBO | Isocaloric carbohydrate control | None |
| 5 | Nabuco (2019) | Whey protein 35 g/d | Whey | Single-source whey protein supplement | None |
| 5 | Nabuco (2019) | Isocaloric placebo | PBO | Isocaloric control | None |
| 6 | Mobley (2017) | Whey protein 30 g/d | Whey | Single-source whey protein supplement | None |
| 6 | Mobley (2017) | Soy protein 30 g/d | Soy | Single-source soy protein supplement | None |
| 6 | Mobley (2017) | Maltodextrin placebo | PBO | Isocaloric carbohydrate control | None |
| 7 | Kerksick (2006) | Whey protein 40 g/d | Whey | Whey protein isolate as primary protein source | None |
| 7 | Kerksick (2006) | Whey + casein + BCAA 40 g/d | Blend | Multi-source blend; no single source >60% | BCAA co-supplement |
| 7 | Kerksick (2006) | Whey + casein 40 g/d | Blend | Multi-source protein blend | None |
| 7 | Kerksick (2006) | Carbohydrate placebo | PBO | Isocaloric carbohydrate control | None |
| 8 | Cribb (2006) | Whey protein 75 g/d | Whey | Single-source whey protein supplement | None |
| 8 | Cribb (2006) | Casein protein 75 g/d | Casein | Single-source casein protein supplement | None |
| 8 | Cribb (2006) | Carbohydrate placebo | PBO | Isocaloric carbohydrate control | None |
| 9 | Willoughby (2007) | Whey + amino acids 20 g/d | Whey | Whey as primary protein; leucine added as enhancer | Added amino acid |
| 9 | Willoughby (2007) | Carbohydrate placebo | PBO | Isocaloric carbohydrate control | None |
| 10 | Park (2019) | Whey blend 40 g/d | Whey | Whey-based protein blend supplement | None |
| 10 | Park (2019) | Carbohydrate placebo | PBO | Isocaloric carbohydrate control | None |
| 11 | Weinheimer (2012) | Whey protein 20 g/d | Whey | Single-source whey protein supplement | None |
| 11 | Weinheimer (2012) | Carbohydrate placebo | PBO | Isocaloric carbohydrate control | None |
| 12 | Snijders (2015) | Casein protein 28 g/d | Casein | Single-source casein supplement (pre-sleep) | None |
| 12 | Snijders (2015) | Non-caloric placebo | PBO | Non-caloric control | None |
| 13 | Demling (2000) | Casein protein 60 g/d | Casein | Single-source casein supplement | None |
| 13 | Demling (2000) | Whey protein 60 g/d | Whey | Single-source whey protein supplement | None |
| 14 | Wilborn (2013) | Whey protein 24 g/d | Whey | Single-source whey protein supplement | None |
| 14 | Wilborn (2013) | Casein protein 24 g/d | Casein | Single-source casein supplement | None |
| 15 | Joy (2018) | Casein protein 35 g/d | Casein | Single-source casein supplement | None |
| 15 | Joy (2018) | Carbohydrate placebo | PBO | Isocaloric carbohydrate control | None |
| 16 | Fabre (2014) | Casein protein 30 g/d | Casein | Single-source casein supplement | None |
| 16 | Fabre (2014) | Milk protein 30 g/d | Milk | Whole milk protein supplement | None |
| 16 | Fabre (2014) | Non-caloric placebo | PBO | Non-caloric control | None |
| 17 | Candow (2006) | Soy protein 48 g/d | Soy | Single-source soy protein supplement | None |
| 17 | Candow (2006) | Whey protein 48 g/d | Whey | Single-source whey protein supplement | None |
| 17 | Candow (2006) | Carbohydrate placebo | PBO | Isocaloric carbohydrate control | None |
| 18 | Hartman (2007) | Milk 18 g/d | Milk | Fat-free fluid milk | None |
| 18 | Hartman (2007) | Soy protein 18 g/d | Soy | Isonitrogenous soy beverage | None |
| 18 | Hartman (2007) | Carbohydrate placebo | PBO | Isocaloric carbohydrate control | None |
| 19 | Babault (2015) | Pea protein 50 g/d | Pea | Single-source pea protein isolate | None |
| 19 | Babault (2015) | Whey protein 50 g/d | Whey | Single-source whey protein supplement | None |
| 19 | Babault (2015) | Placebo | PBO | Placebo control | None |
| 20 | Banaszek (2019) | Pea protein 24 g/d | Pea | Single-source pea protein supplement | None |
| 20 | Banaszek (2019) | Whey protein 24 g/d | Whey | Single-source whey protein supplement | None |
| 21 | Nieman (2024) | Pea protein 25 g/d | Pea | Single-source pea protein supplement | None |
| 21 | Nieman (2024) | Soy protein 25 g/d | Soy | Single-source soy protein supplement | None |
| 21 | Nieman (2024) | Whey protein 25 g/d | Whey | Single-source whey protein supplement | None |
| 21 | Nieman (2024) | Maltodextrin placebo | PBO | Isocaloric carbohydrate control | None |
| 22 | Joy (2013) | Rice protein 48 g/d | Rice | Single-source rice protein supplement | None |
| 22 | Joy (2013) | Whey protein 48 g/d | Whey | Single-source whey protein supplement | None |
| 22 | Joy (2013) | Carbohydrate placebo | PBO | Isocaloric carbohydrate control | None |
| 23 | Moon (2020) | Rice protein 24 g/d | Rice | Single-source rice protein supplement | None |
| 23 | Moon (2020) | Whey protein 24 g/d | Whey | Single-source whey protein supplement | None |
| 23 | Moon (2020) | Carbohydrate placebo | PBO | Isocaloric carbohydrate control | None |
| 24 | Josse (2010) | Milk 25 g/d | Milk | Fat-free fluid milk | None |
| 24 | Josse (2010) | Carbohydrate placebo | PBO | Isocaloric carbohydrate control | None |
| 25 | Rankin (2004) | Milk 20 g/d | Milk | Chocolate milk | None |
| 25 | Rankin (2004) | Carbohydrate placebo | PBO | Isocaloric carbohydrate control | None |
| 26 | Reidy (2016) | Protein blend 22 g/d | Blend | Whey + soy + casein blend | None |
| 26 | Reidy (2016) | Soy protein 22 g/d | Soy | Single-source soy protein supplement | None |
| 26 | Reidy (2016) | Whey protein 22 g/d | Whey | Single-source whey protein supplement | None |
| 26 | Reidy (2016) | Carbohydrate placebo | PBO | Isocaloric carbohydrate control | None |
| 27 | Kerksick (2007) | Whey + casein + creatine 40 g/d | Blend | Multi-source protein blend with creatine | Creatine co-supplement |
| 27 | Kerksick (2007) | Whey + creatine 40 g/d | Whey | Whey as primary protein with creatine | Creatine co-supplement |
| 27 | Kerksick (2007) | Carbohydrate placebo | PBO | Isocaloric carbohydrate control | None |
| 28 | Antonio (2014) | Whey protein (high-protein diet) 75 g/d | Whey | Supplemental whey protein powder | High-protein diet (4.4 g/kg/d total) |
| 28 | Antonio (2014) | Habitual diet control | PBO | Habitual diet control | None |
| 29 | Tieland (2012) | Casein protein 30 g/d | Casein | Single-source casein supplement | None |
| 29 | Tieland (2012) | Isocaloric placebo | PBO | Isocaloric control | None |
| 30 | Leenders (2013) | Casein protein 15 g/d | Casein | Single-source casein supplement | None |
| 30 | Leenders (2013) | Non-caloric placebo | PBO | Non-caloric control | None |
| 31 | Verdijk (2009) | Casein protein 20 g/d | Casein | Single-source casein supplement | None |
| 31 | Verdijk (2009) | Isocaloric placebo | PBO | Isocaloric control | None |
| 32 | Chale (2013) | Whey protein 40 g/d | Whey | Single-source whey protein supplement | None |
| 32 | Chale (2013) | Isocaloric control | PBO | Isocaloric control | None |
| 33 | Andersen (2005) | Protein blend 25 g/d | Blend | Multi-source protein blend | None |
| 33 | Andersen (2005) | Carbohydrate placebo | PBO | Isocaloric carbohydrate control | None |
| 34 | Holm (2008) | Milk protein 10 g/d | Milk | Milk protein supplement | None |
| 34 | Holm (2008) | No supplement | PBO | No supplement control | None |
| 35 | Weisgarber (2012) | Whey protein 30 g/d | Whey | Single-source whey protein supplement | None |
| 35 | Weisgarber (2012) | Carbohydrate placebo | PBO | Isocaloric carbohydrate control | None |
| 36 | Haub (2002) | Soy protein 30 g/d | Soy | Single-source soy protein supplement | None |
| 36 | Haub (2002) | Habitual diet control | PBO | Habitual diet control | None |

Note: Co-intervention flags indicate arms excluded in the sensitivity analysis (Section 3.12). Arms flagged with BCAA co-supplement, Added amino acid, Creatine co-supplement, or High-protein diet were removed; studies left with fewer than two arms after exclusion were also removed entirely.

**Supplementary Table S7. Distribution of key effect modifiers across treatment nodes**

| **Characteristic** | **Whey (k=24)** | **Casein (k=9)** | **Soy (k=7)** | **Pea (k=3)** | **Rice (k=2)** | **Milk (k=5)** | **Blend (k=5)** |
| --- | --- | --- | --- | --- | --- | --- | --- |
| Number of arms | 26 | 11 | 8 | 4 | 4 | 6 | 6 |
| Total participants (N) | 420 | 166 | 120 | 83 | 24 | 77 | 77 |
| Mean age (years) | 34.1 | 48.8 | 28.5 | 27.1 | 23.0 | 33.8 | 29.4 |
| Male (%) | 75 | 70 | 88 | 83 | 100 | 69 | 100 |
| Trained (%) | 40 | 29 | 18 | 10 | 100 | 29 | 66 |
| Mean supplement dose (g/d) | 37.3 | 30.7 | 26.8 | 41.2 | 36.0 | 20.8 | 33.6 |
| Mean intervention duration (weeks) | 11.4 | 15.9 | 11.0 | 11.6 | 8.0 | 11.2 | 10.3 |
| DXA use (%) | 79 | 75 | 100 | 25 | 100 | 58 | 100 |
| Baseline dietary protein intake (g/kg/d) | NR | NR | NR | NR | NR | NR | NR |

Note: k = number of studies contributing at least one arm to the node (includes multi-arm trials). Values are sample-size-weighted means across all arms assigned to each node. NR = not consistently reported across the included studies; fewer than half of the included trials reported baseline habitual dietary protein intake, precluding summary by node.

**Supplementary Table S8. League table with 95% credible intervals for lean body mass (kg)**

|  | **Whey (0.91)** | **Milk (0.78)** | **Casein (0.72)** | **Rice (0.65)** | **Blend (0.48)** | **Soy (0.40)** | **Pea (0.35)** | **PBO (0.01)** |
| --- | --- | --- | --- | --- | --- | --- | --- | --- |
| **Whey** | — | 0.06 [−0.74, 0.87] | 0.32 [−0.32, 0.96] | 0.28 [−0.91, 1.46] | 0.62 [−0.14, 1.38] | 0.69 [0.07, 1.33]* | 0.76 [−0.06, 1.60] | 1.24 [0.61, 1.87]* |
| **Milk** | −0.06 [−0.87, 0.74] | — | 0.26 [−0.62, 1.13] | 0.22 [−1.20, 1.61] | 0.56 [−0.47, 1.57] | 0.63 [−0.26, 1.53] | 0.70 [−0.41, 1.80] | 1.18 [0.22, 2.14]* |
| **Casein** | −0.32 [−0.96, 0.32] | −0.26 [−1.13, 0.62] | — | −0.04 [−1.37, 1.26] | 0.30 [−0.64, 1.21] | 0.37 [−0.44, 1.19] | 0.44 [−0.55, 1.44] | 0.92 [0.18, 1.66]* |
| **Rice** | −0.28 [−1.46, 0.91] | −0.22 [−1.61, 1.20] | 0.04 [−1.26, 1.37] | — | 0.34 [−1.06, 1.71] | 0.41 [−0.89, 1.73] | 0.48 [−0.90, 1.94] | 0.96 [−0.12, 2.04] |
| **Blend** | −0.62 [−1.38, 0.14] | −0.56 [−1.57, 0.47] | −0.30 [−1.21, 0.64] | −0.34 [−1.71, 1.06] | — | 0.07 [−0.99, 0.81] | 0.14 [−1.24, 0.95] | 0.62 [−0.34, 1.58] |
| **Soy** | −0.69 [−1.33, −0.07]* | −0.63 [−1.53, 0.26] | −0.37 [−1.19, 0.44] | −0.41 [−1.73, 0.89] | −0.07 [−0.81, 0.99] | — | 0.07 [−0.90, 1.03] | 0.55 [−0.15, 1.25] |
| **Pea** | −0.76 [−1.60, 0.06] | −0.70 [−1.80, 0.41] | −0.44 [−1.44, 0.55] | −0.48 [−1.94, 0.90] | −0.14 [−0.95, 1.24] | −0.07 [−1.03, 0.90] | — | 0.48 [−0.20, 1.16] |
| **PBO** | −1.24 [−1.87, −0.61]* | −1.18 [−2.14, −0.22]* | −0.92 [−1.66, −0.18]* | −0.96 [−2.04, 0.12] | −0.62 [−1.58, 0.34] | −0.55 [−1.25, 0.15] | −0.48 [−1.16, 0.20] | — |

Note: Each cell shows the mean difference in LBM change (kg) [95% credible interval] between the row treatment and the column treatment. Positive values favor the row treatment. * = 95% CrI excludes zero. Diagonal cells show the treatment name and SUCRA value in parentheses. Values in the rightmost column (vs. PBO) correspond to those reported in Table 2 and Figure 4 of the main manuscript.

# Supplementary Figure S1: Risk of bias summary (RoB 2.0 traffic-light plot and bar chart)

# Supplementary Figure S2: MCMC trace plots and density plots for LBM model

# Supplementary Figure S3: Forest plot for muscular strength (1-RM) outcome

# Supplementary Figure S4: Contribution matrix for the LBM network

# Supplementary Figure S5: Net heat plot for inconsistency assessment

# Supplementary Figure S6: Leave-one-out sensitivity analysis (SUCRA variation plot)

# Supplementary Figure S7: Age-stratified dose--response curves (younger vs. older adults)

# Supplementary Figure S8: Prior sensitivity analysis (vague vs. informative prior posterior comparison)

# Supplementary Figure S9. Cluster ranking plot for the simultaneous evaluation of the outcomes of lean body mass and muscular strength. Each point represents the type of protein. The size of the points corresponds to the total number of trial arms. The treatments in the upper-right quadrant indicate good performance.
